# Rapid and long-lasting improvements in neural discrimination of acoustic signals with passive familiarization

**DOI:** 10.1371/journal.pone.0221819

**Published:** 2019-08-29

**Authors:** Efe Soyman, David S. Vicario

**Affiliations:** Department of Psychology, Rutgers, the State University of New Jersey, New Brunswick, New Jersey, United States of America; Claremont Colleges, UNITED STATES

## Abstract

Sensory representations in the adult brain must undergo dynamic changes to adapt to the complexity of the external world. This study investigated how passive exposure to novel sounds modifies neural representations to facilitate recognition and discrimination, using the zebra finch model organism. The neural responses in an auditory structure in the zebra finch brain, Caudal Medial Nidopallium (NCM), undergo a long-term form of adaptation with repeated stimulus presentation, providing an excellent substrate to probe the neural underpinnings of adaptive sensory representations. In Experiment 1, electrophysiological activity in NCM was recorded under passive listening conditions as novel natural vocalizations were familiarized through playback. Neural decoding of stimuli using the temporal profiles of both single-unit and multi-unit responses improved dramatically during the first few stimulus presentations. During subsequent encounters, these signals were recognized after hearing fewer initial acoustic features. Remarkably, the accuracy of neural decoding was higher when different stimuli were heard in separate blocks compared to when they were presented randomly in a shuffled sequence. NCM neurons with narrow spike waveforms generally yielded higher neural decoding accuracy than wide spike neurons, but the rate at which these accuracies improved with passive exposure was comparable between the two neuron types. Experiment 2 supported and extended these findings by showing that the rapid gains in neural decoding of novel vocalizations with passive familiarization were long-lasting, maintained for 20 hours after the initial encounter, in multi-unit responses. Taken together, these findings provide valuable insights into the mechanisms by which the nervous system dynamically modulates sensory representations to improve discrimination of novel complex signals over short and long timescales. Similar mechanisms may also be engaged during processing of human speech signals, and thus may have potential translational relevance for elucidating the neural basis of speech comprehension difficulties.

## Introduction

Throughout life, organisms encounter novel sensory signals, whether they are unfamiliar patterns drawn from the distribution of familiar stimulus statistics or instances of completely novel stimulus features. To cope with this problem, sensory systems retain a considerable degree of plasticity in the adult brain [1,2]. This plasticity works at multiple timescales, from rapid, transient dynamics to signals embedded in a context or sequence [3,4] to long-lasting changes in neural representations [5]. The neural mechanisms by which sensory experiences induce these changes remain largely unknown. This study approaches this problem by examining how passive exposure to novel sounds modifies neural representations to facilitate stimulus recognition, using the zebra finch (*Taeniopygia guttata*) model organism.

Zebra finches use complex, learned acoustic signals for social communication with many parallels to human speech, such as individual recognition [6] and categorical perception [7]. Furthermore, the neural responses in the Caudal Medial Nidopallium (NCM), an auditory structure in the zebra finch forebrain, undergo a long-term form of adaptation with repeated stimulus presentation [8–13], providing an excellent substrate to probe the neural underpinnings of dynamic sensory representations. NCM is believed to be analogous to the secondary auditory cortex in the mammalian brain [14] and consists of functionally heterogeneous neurons that reliably represent different features of the individually-specific complex vocalizations of other conspecifics at fine temporal resolutions [15]. Electrophysiological studies investigating the phenomenon of adaptation in NCM documented that neural responses to initial presentations of a novel stimulus are robust, but gradually decrease with repeated presentation [8]. When another novel sound is then presented, the initial responses are again robust and adapt independently from the first stimulus. This process is stimulus-specific, because when the first stimulus is presented again after several presentations of other sounds, neural responses do not start at initial high magnitudes, but remain at adapted levels [8]. In this sense, the adaptation process forms long-term neuronal memories that can be detected hours to days after the initial induction [8–10].

What remains still unknown is how the changes in neural representations with adaptation affect the recognition of novel acoustic signals. The form of adaptation documented in the songbird auditory system fundamentally differs from the process of stimulus-specific adaptation (SSA) extensively studied in the mammalian auditory system using the classical oddball paradigm [16]. SSA is transient and only reflects stimulus statistics over a relatively short timescale (seconds), whereas adaptation to specific stimuli in songbird NCM can last several days [10] or more [17]. SSA has been proposed to improve the detection of a rare stimulus in a series of presentations of a common stimulus; however, neural discrimination can only be assessed between the common and the rare stimuli using the experimental and analytical methods described in studies of the mammalian SSA [18]. This is completely different from the neural recognition changes investigated in this study, which compares the temporal profiles of neural responses to stimuli with equal probabilities of occurrence as they all go from being completely novel to being familiar through repeated passive exposure. Very recently, an adaptation process showing similar long-term dynamics as in the songbird NCM has been reported in the secondary, but not in the primary, auditory cortex of ferrets [19]. Furthermore, mutual information between stimulus identities and neural responses increased from the first to the second half of the presentations of these complex novel signals. In the human speech processing literature, there is compelling evidence that passive auditory exposure to distorted speech signals, such as foreign-accented [20,21] or dysarthric speech [22], improves behavioral measures of recognition and comprehension [23]. These findings are in line with the idea that dynamic sensory representations are updated accordingly during passive exposure to improve the mapping between the incoming sensory signals and learned linguistic categories, such as phonemes. The small number of studies investigating the neural basis of these adaptive changes has shown a rather complicated picture. Several auditory, motor, and sensorimotor brain regions, such as the auditory association cortex [24] and the left ventral premotor cortex [25], were found to be involved in the improvements in comprehension of distorted speech signals following passive familiarization [23]. However, due to temporal resolution limitations, these functional magnetic resonance imaging (fMRI) studies were not able to provide any information as to how neural representations of these distorted speech signals changed as they become familiar through passive exposure.

To address this gap in our knowledge, the present study investigated how changes in the fine temporal profiles of neural responses in the zebra finch NCM with repeated stimulus exposure affect neural recognition of novel acoustic signals. In Experiment 1, we tested the hypothesis that stimulus discrimination and decoding improve with ongoing adaptation as novel signals become familiar, as reflected in the dynamic temporal profiles of neural responses. The second major goal of Experiment 1 was to assess whether the rapid changes in temporal profiles of neural responses with stimulus repetition reflect exposure not only to the target stimulus itself, but also to other signals embedded in a sequence, in such a way as to improve the neural contrast between them. In Experiment 2, we moved from the immediate to the long-lasting effects of passive stimulus exposure, and tested the hypothesis that improved neural recognition, induced during initial presentation to a set of novel signals, is maintained and can be detected during testing 20 hours after that exposure. The results demonstrate that the adaptation process is associated with improved stimulus discrimination at the neural level, document the dynamics of this improvement under different conditions (Experiment 1), and show that this improvement is long-lasting (Experiment 2). The findings thus add to our understanding of the mechanisms of passive familiarization and statistical learning.

## Results

### Experiment 1

**Fig 1:**
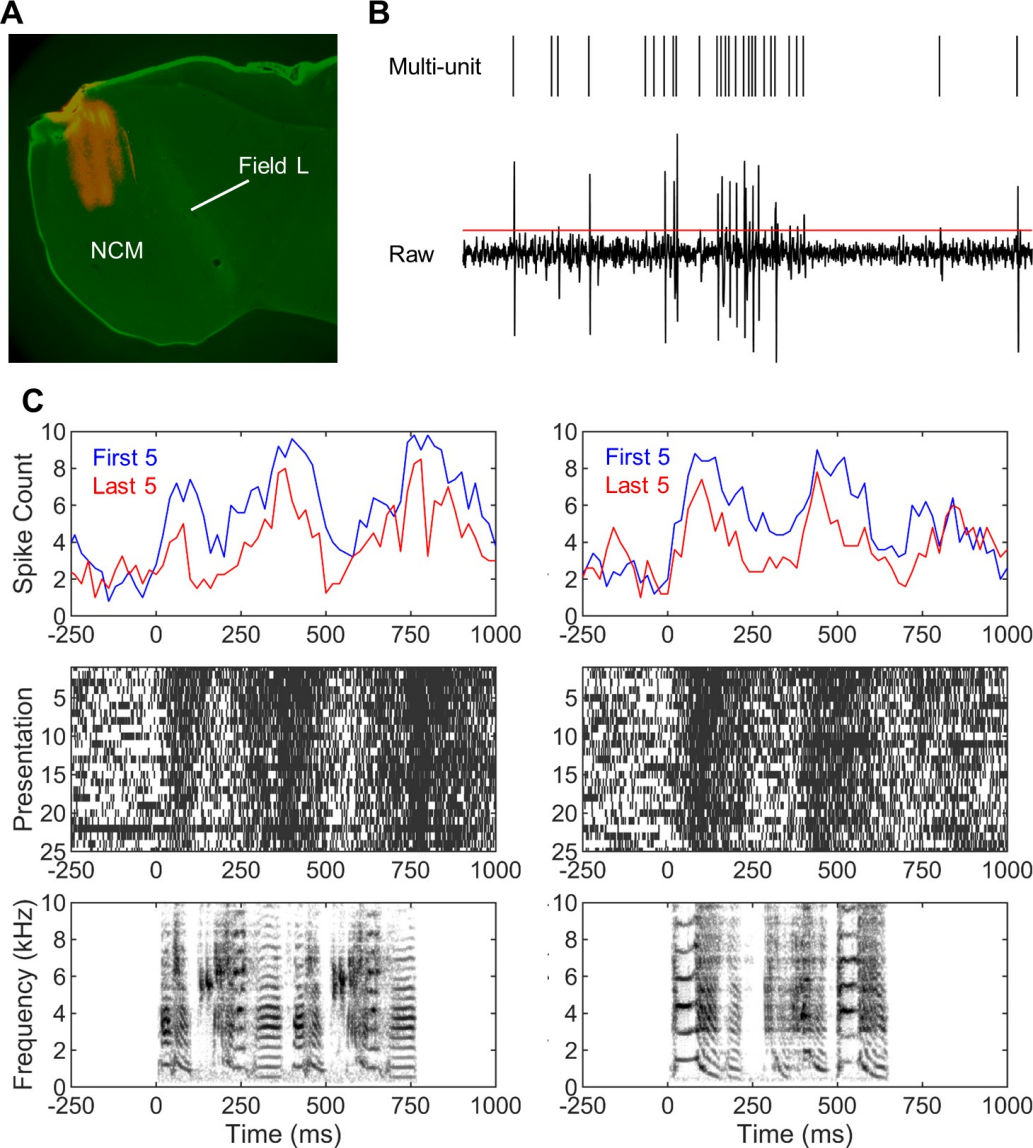
NCM and adaptation. (A) Recording sites were histologically verified to be in NCM via DiI labeling (red). Top is dorsal, right is anterior. (B) Raw electrophysiological recordings were thresholded at 2 standard deviations of the whole recording and the peaks of positive threshold-crossings were marked with timestamps to extract multi-unit spike trains. (C) The peristimulus time histograms (top panel) and raster plots (middle panel) depicting an example of multi-unit activity in response to two songs (bottom panel) clearly show the effect of adaptation on the temporal profiles of neural responses over 25 stimulus presentations. Note that the temporal profiles of neural activity in response to the two stimuli are more dissimilar from each other during the last 5 than the first 5 stimulus presentations.

To assess how passive exposure to novel acoustic signals affect their neural recognition as they become familiar, 16 male zebra finches were presented with 25 repetitions of each of 8 novel conspecific songs during awake, restrained electrophysiological recordings bilaterally from NCM. For half of the birds, the experimental stimuli were presented in a blocked order, while a shuffled (randomized) sequence was used for the other half. There were 184 multi-unit sites recorded with the blocked sequence and 152 multi-unit sites with the shuffled sequence, all histologically verified to be in NCM (**Fig 1A**). In addition, 106 single-units in the blocked and 113 single-units in the shuffled sequence were sorted from these multi-unit recordings. Preliminary analyses did not show systematic hemispheric differences in any of the basic response properties or the neural discrimination metrics in either the blocked or the shuffled sequence. Thus, data from the two hemispheres were combined for all subsequent analyses. Unless specified as single-units, all analyses were based on multi-unit responses.

#### Adaptation occurs faster in the blocked than in the shuffled sequence

**Fig 2:**
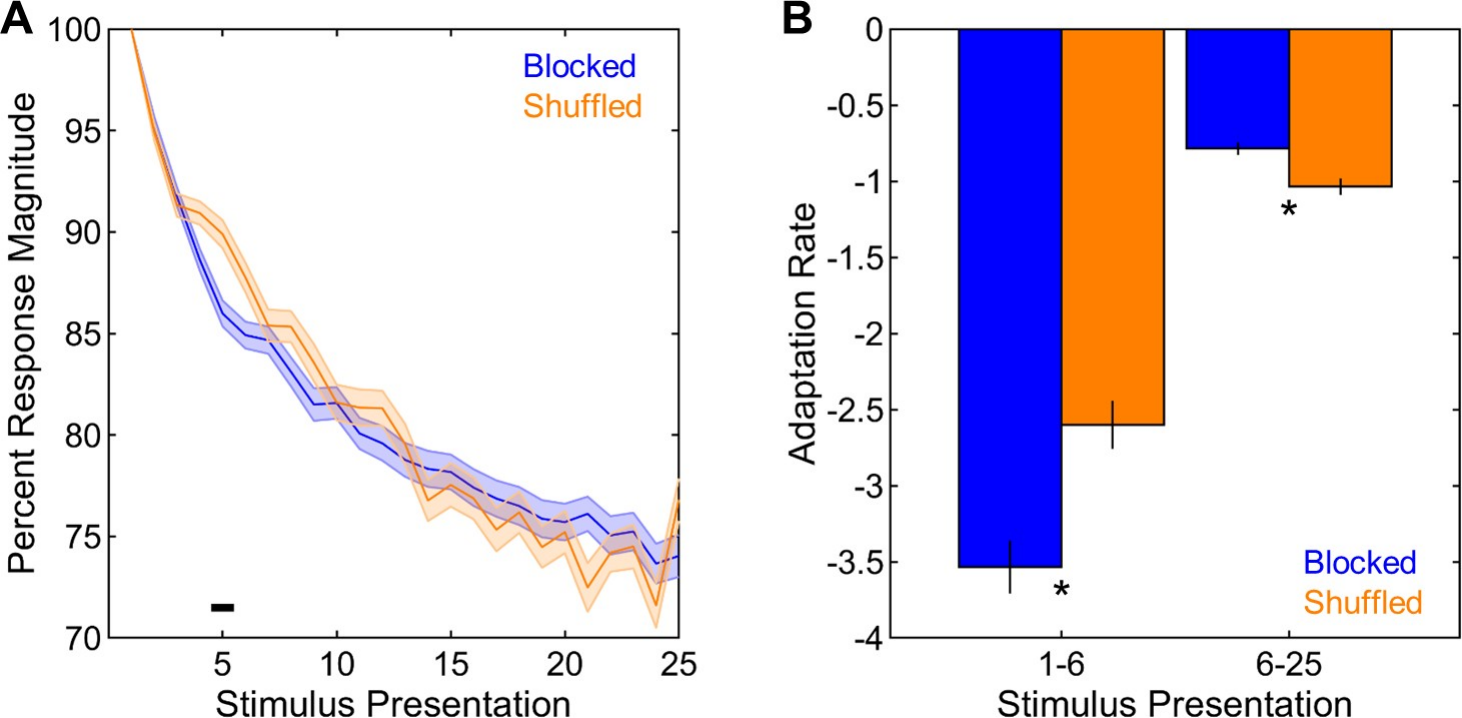
Adaptation in blocked and shuffled sequences. (A) The decrease in response magnitude (calculated as a percent of response to the first presentation) across stimulus presentations was comparable between the two sequences. (B) Adaptation rates (see Methods) in the blocked sequence were more negative for presentations 1–6 and less negative for presentations 6–25 than those in the shuffled sequence. Shadings and error bars indicate SEMs. Horizontal black bar and asterisks denote significant differences.

First, the magnitudes of stimulus-driven neural responses across stimulus presentations were compared between the blocked and shuffled sequences. Overall, percent response magnitudes gradually decreased with stimulus presentation (F(23,7682) = 571.65, p < 0.001, **Fig 2A**), as expected from the well-known process of adaptation in NCM [8–10]. The overall magnitudes of responses were not different between the blocked and the shuffled sequence (F(1,334) = 0.05, p = 0.821); however, the ways in which these responses adapted differed between the two sequences (F(23,7682) = 12.68, p < 0.001, **Fig 2A**). To analyze these differences further in detail, the rates of adaptation were quantified for stimulus presentations 1 through 6 and 6 through 25 (hereafter referred to as 1–6 and 6–25, respectively) separately, as in previous studies [17,26]. Adaptation was generally stronger during presentations 1–6 than 6–25 (F(1,334) = 428.09, p < 0.001, **Fig 2B**) and in the blocked than in the shuffled sequence (F(1,334) = 5.82, p = 0.016, **Fig 2B**). More importantly, there was an interaction between sequence and stimulus presentation (F(1,334) = 32.22, p < 0.001, **Fig 2B**), such that the blocked sequence had steeper adaptation rates than the shuffled sequence during presentations 1–6 (t(334) = 3.90, p < 0.001), while the reverse was true for presentations 6–25 (t(334) = 3.77, p < 0.001). Looking at the interaction from the other perspective, adaptation was stronger during presentations 1–6 than during presentations 6–25 in both the blocked (t(183) = 18.64, p < 0.001) and the shuffled sequence (t(151) = 10.86, p < 0.001). Taken together, these findings indicate that adaptation occurs slightly faster in the blocked than in the shuffled sequence.

#### Neural decoding accuracy rapidly improves with repeated stimulus presentation

Next, we investigated how the neural recognition of novel stimuli changed as they become familiar through passive repeated exposure. Visual examination of neural response magnitudes across stimulus presentations revealed that adaptation was not uniform along the stimulus duration; instead, it was very strong at certain time points, but very little or absent at others (**Fig 1C**). Furthermore, these patterns differed between different stimuli. Taken together, these observations led to the working hypothesis that the temporal profiles of neural responses underwent specific changes with repeated exposure to enhance the neural contrasts between different novel signals. This hypothesis was assessed by utilizing a neural decoding method based on the temporal profiles of neural responses. Briefly, neural responses were binned at 10-ms temporal resolution and standardized by taking the z-score of each bin for each stimulus presentation; stimuli lasted ~750ms, thus there were 75 bins across the stimulus duration. This procedure assured that the changes in neural discrimination metrics would be solely due to the changes in the temporal profile in neural activity over the stimulus duration, and not due to the global changes in the magnitudes of neural responses across stimulus presentations. Neural dissimilarity was then quantified by calculating the Euclidean distance between neural response profiles to different stimuli for the same site. These pairwise dissimilarities were used to decode stimulus identities by averaging across presentations of each stimulus and classifying the responses to the least dissimilar stimulus average. To assess how neural decoding evolved over the stimulus duration, these calculations were carried out in an iterative fashion by progressively increasing the number of time bins that went into the calculation starting from the stimulus onset. For decoding at each cumulative bin, the probability of correct decoding was calculated by calculating the fraction of the 8 stimuli that were correctly classified for a given stimulus presentation.

**Fig 3:**
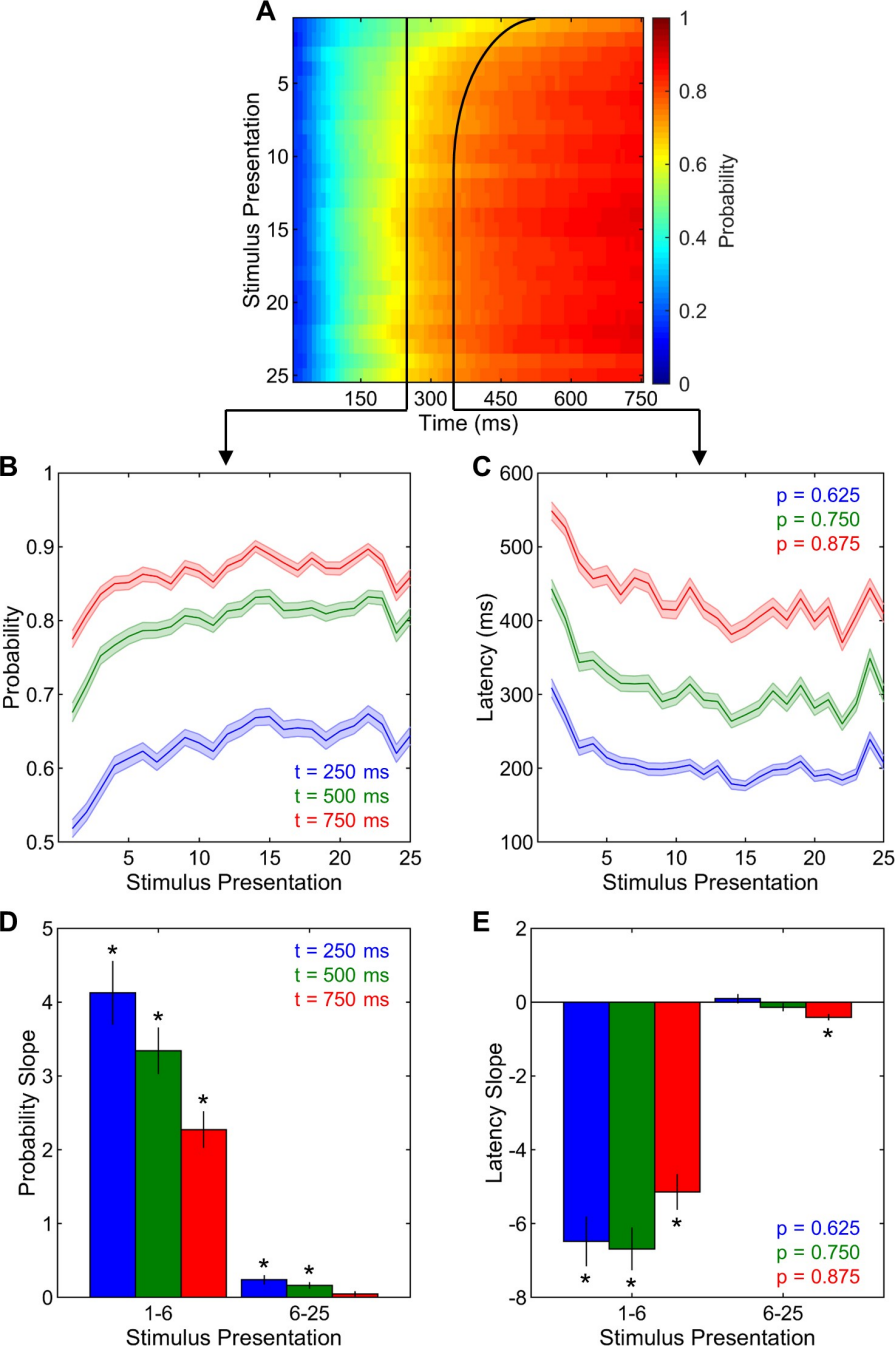
Neural decoding. (A) Neural decoding accuracies were analyzed as the probability of correct decoding across time points along the stimulus duration and stimulus presentations. The black arrows schematically indicate the subsequent time point (left) and probability level (right) analyses. (B) Correct neural decoding probabilities at all selected time points increased with stimulus presentations. (C) Correct neural decoding latencies at all selected probability levels decreased with stimulus presentations. (D) The slopes of correct neural decoding probabilities were higher than zero at all time points for presentations 1–6 and at the 250 and 500-ms time points for presentations 6–25. (E) The slopes of correct neural decoding latencies were lower than zero at all probability levels for presentations 1–6 and at the 0.875 probability level for presentations 6–25. Shadings and error bars indicate SEMs. Asterisks denote significant differences from zero.

For the first set of analyses, the blocked and shuffled sequences were combined to focus solely on how neural decoding accuracies changed with stimulus presentation. **Fig 3A** depicts the correct neural decoding probabilities across time points along the stimulus duration and across stimulus presentations. Visual examination of this figure clearly indicates that the probability of correct neural decoding increases with time along the stimulus duration and also with stimulus presentation. These improvements seem to occur during the first 10 to 15 presentations and remain at asymptotic levels afterwards. To analyze these changes in detail, neural decoding results were dissected in two different ways. One way was to select different time points along the stimulus duration and examine how correct neural decoding probabilities changed with stimulus presentation at those time points. This analysis was conducted at 3 different time points: 250, 500, and 750 ms. At all 3 time points, there were significant increases in correct neural decoding probabilities across stimulus presentations (all F(24,8040) > 22.70, p < 0.001, **Fig 3B**). These increases were further investigated by fitting regression lines and calculating the normalized slopes of probabilities during stimulus presentations 1–6 and 6–25, separately, in conjunction with the computation of adaptation rates. The slopes of correct neural decoding probabilities at 250 and 500 ms time points were significantly greater than 0 for both presentations 1–6 and 6–25 (all t(335) > 3.62, p < 0.001, **Fig 3D**), indicating significant improvements in neural decoding accuracies with repeated stimulus presentation. At 750 ms time point, the slopes were significantly greater than 0 for presentations 1–6 (t(335) = 9.18, p < 0.001, **Fig 3D**), but not for presentations 6–25 (t(335) = 1.38, p = 0.167). At all 3 time points, the slopes were significantly higher for presentations 1–6 than for presentations 6–25 (all t(335) > 8.84, p < 0.001, **Fig 3D**), indicating that the improvements in correct neural decoding probabilities were much more pronounced during early stimulus presentations than the later ones. Taken together, these findings clearly support our main hypothesis that novel auditory signals can be neurally decoded at a greater and greater accuracy as those signals become more and more familiar through passive repeated exposure. Furthermore, these improvements are very robust during the initial and much more modest during the later stimulus presentations.

Another way of dissecting the neural decoding results in **Fig 3A**, rather than fixing time and allowing probabilities to vary, was to select different correct decoding probability levels and examine how latencies along the stimulus duration to reach those probability levels changed with stimulus presentation. This analysis was conducted for 3 different probability levels: 0.625 (5 out of 8 stimuli correctly classified), 0.750 (6 out of 8), and 0.875 (7 out of 8). There were significant reductions in correct decoding latencies for all 3 probability levels (all F(24,8040) > 18.91, p < 0.001, **Fig 3C**). These reductions were further investigated by analyzing the slopes of correct neural decoding latencies during stimulus presentations 1 to 6 and 6 to 25, separately. For all 3 probability levels, the slopes of correct neural decoding latencies for presentations 1–6 were significantly lower than zero (all t(335) > 9.60, p < 0.001, **Fig 3E**), indicating significant reductions with repeated stimulus presentation in the latencies to reach the same neural decoding accuracy level. The slopes for presentations 6–25 were significantly lower than 0 for the 0.875 probability level (t(335) = 4,61, p < 0.001, **Fig 3E**), but not for the 0.750 or the 0.675 probability levels (both t(335) < 1.28, p > 0.205). For all 3 probability levels, the slopes were significantly lower for presentations 1–6 than for presentations 6–25 (all t(335) > 9.33, p < 0.001, **Fig 3E**), suggesting that the reductions in correct neural decoding latencies were much more pronounced during early stimulus presentations than the later ones. To sum, these results strongly demonstrate that novel auditory signals can be neurally decoded at a given confidence level sooner and sooner along the stimulus duration as those signals become more and more familiar through passive repeated exposure. Moreover, these changes in latencies are much more pronounced during the initial exposures and very modest or even absent thereafter.

#### Neural responses become more differentiated for different stimuli and more consistent for the same stimulus over initial stimulus presentations

**Fig 4:**
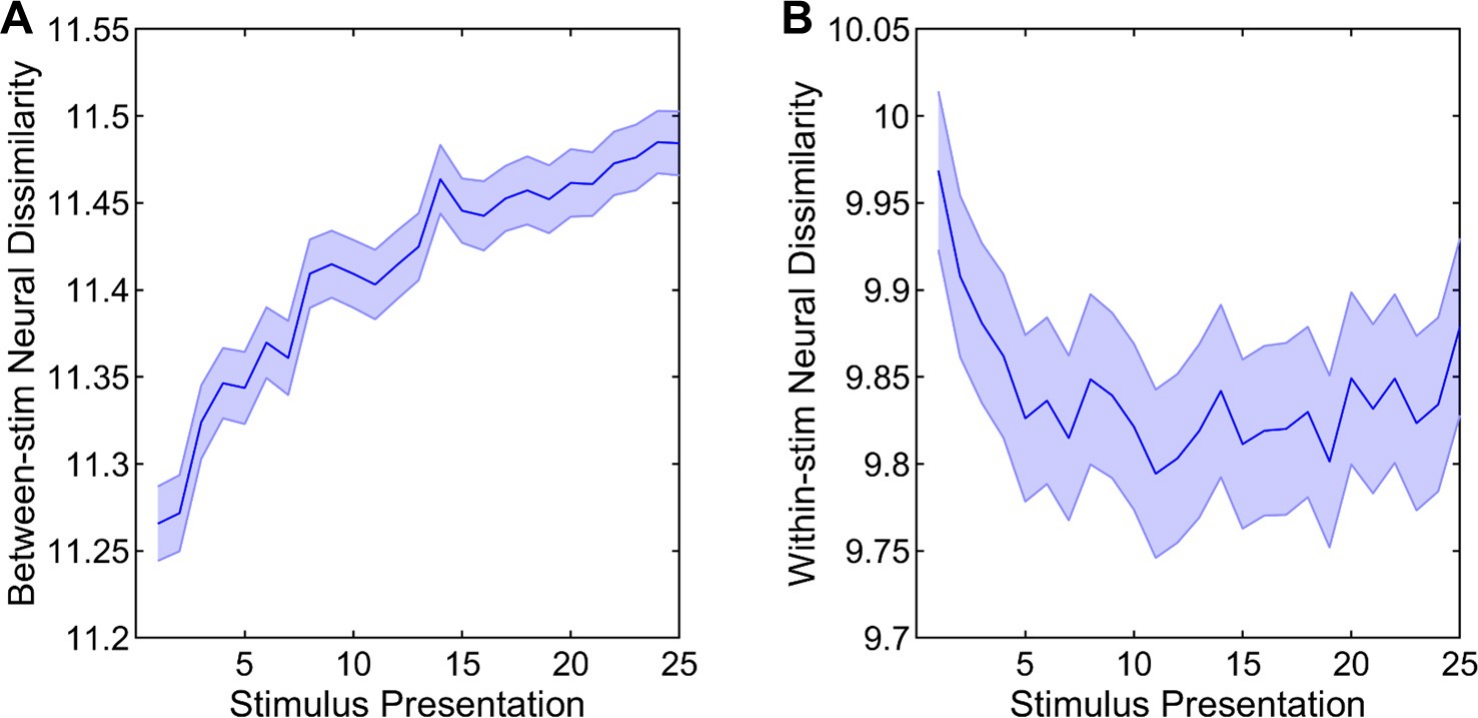
Neural dissimilarities. (A) Between-stimulus neural dissimilarities increased with stimulus presentation during both presentations 1–6 and 6–25. (B) Within-stimulus neural dissimilarities decreased during presentations 1–6 and then slightly increased during presentations 6–25. Shadings indicate SEMs.

Having confirmed the robust improvements in neural recognition of novel stimuli with repeated stimulus presentation, the specific dynamics underlying these changes were further investigated in detail. To do this, neural dissimilarities were calculated in two different ways. One way was to calculate the neural dissimilarities between the presentations of different stimuli, which quantify the differences between the temporal profiles of neural responses to different stimuli. These between-stimulus neural dissimilarities significantly increased with stimulus presentation (F(24,8040) = 98.83, p < 0.001, **Fig 4A**). These improvements were further investigated by the analyzing the slopes of between-stimulus neural dissimilarities for presentations 1–6 and 6–25, separately. The slopes were significantly higher than 0 for both presentations 1–6 and 6–25 (both t(335) > 14.00, p < 0.001). In addition, the slopes for presentations 1–6 were significantly higher than for presentations 6–25 (t(335) = 10.48, p < 0.001). Thus, these findings indicate that the neural representations of different novel stimuli become more and more differentiated from each other as those stimuli become more and more familiar with repeated stimulus presentation and these changes are much more robust during the early as compared to the later stimulus exposures.

Another way to assess neural dissimilarities was to calculate them between the different presentations of the same stimulus, which quantifies how different the temporal profiles of neural responses to a given stimulus are. These within-stimulus neural dissimilarities significantly changed with stimulus presentation (F(24,8040) = 20.42, p < 0.001, **Fig 4B**). The examination of these changes by the analysis of slopes for presentations 1–6 and 6–25 indicated that the slopes were significantly lower than 0 for presentations 1–6 (t(335) = 12.10, p < 0.001), suggesting significant reductions in within-stimulus neural dissimilarities during early stimulus presentations. The slopes for presentations 6–25, on the other hand, were slightly higher than 0 (t(335) = 2.25, p = 0.025), indicating modest increases in within-stimulus neural dissimilarities during later stimulus presentations. Not surprisingly, the slopes were significantly lower for presentations 1–6 than for presentations 6–25 (t(335) = 12.52, p < 0.001). Taken together, these findings indicate that the neural representations of any particular novel stimulus dramatically become more and more consistent during the initial encounters. Puzzlingly, they then slightly lose consistency during later repetitions.

#### Steeper adaptation correlates with stronger improvements in neural decoding accuracy

**Fig 5:**
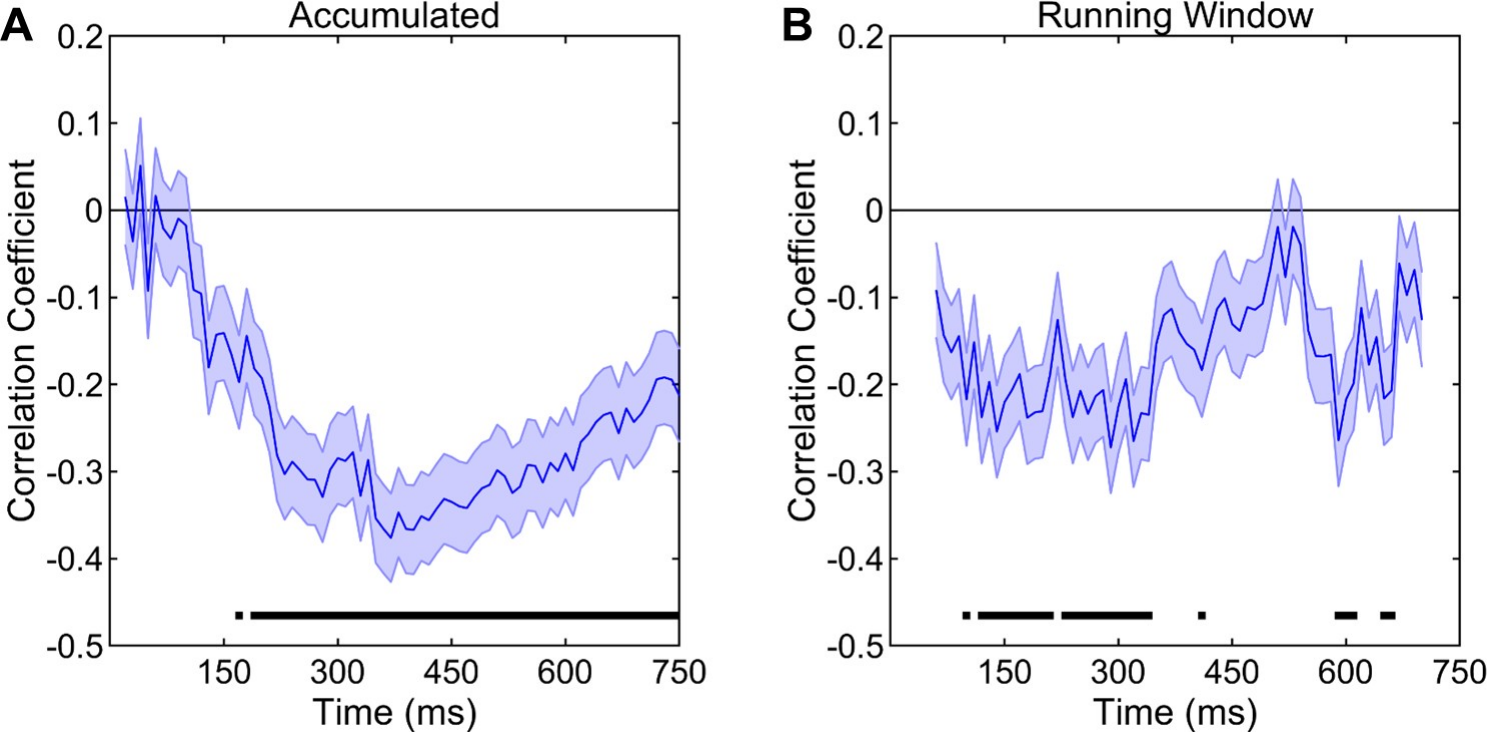
Correlations between adaptation rates and the slopes of correct neural decoding probabilities. (A) The correlations between adaptation rates and the slopes of correct neural decoding probabilities for presentations 1–6 were negative during response windows starting from the stimulus onset and ending at the 17^th^ time bin or further (170–750 ms). (B) When the same relationship between the two variables was assessed using a 110-ms running response window, the correlations were negative for several response windows, majority of which were mostly centered between the 10^th^ and the 30^th^ time bin (100–300 ms). Shadings indicate standard error of correlation coefficients. Horizontal black bars denote significant correlations.

One of our main hypotheses was that the process of adaptation was related to improvements in neural decoding accuracies with repeated stimulus presentations. To test this, both adaptation rates and the slopes of correct neural decoding probabilities were calculated across time points along the stimulus duration in an accumulative fashion, as was done for the neural decoding results described above. Then the correlations between the adaptation rates and probability slopes were analyzed for presentations 1–6 and 6–25 separately. For presentations 1–6, there were significant negative correlations between adaptation rates and the slopes of correct neural decoding probabilities from 170 ms (the 17^th^ time bin) until the end of the stimulus duration at 750 ms (all r(334) < -0.19, p < 0.05/75 for 75 separate time bins, **Fig 5A**). No such relationship was observed for any time bin for presentations 6–25. The negative correlations clearly demonstrate that, during the initial, but not later, stimulus presentations, sites that undergo stronger adaptation (more negative slopes) also display much more pronounced improvements in neural decoding accuracy. This relationship becomes significant only after hearing the initial 170 ms of the stimulus or more and peaks when 350–400 ms of the stimulus has been heard.

The above analysis was conducted by progressively accumulating information from the beginning of the stimulus duration. Significant temporal changes in this analysis suggests that there might be ideal local time windows along the stimulus duration where the relationship between adaptation and decoding improvements would be at maximal levels. To probe this further in detail, adaptation rates and probability slopes were separately calculated again, but this time, instead of accumulating time from the beginning of the stimulus duration, a 110-ms running time window was used. The correlations between the two variables were again analyzed for presentations 1–6 and 6–25 separately. For presentations 1–6, significant negative correlations were observed between adaptation rates and the slopes of correct neural decoding probabilities at several time points starting with the time window centered at the 10^th^ time bin (100 ms, all r(334) < -0.18, p < 0.05/65 for 65 separate time windows, **Fig 5B**). The time interval at which this relationship was the strongest was from 100 ms to 300 ms. This was perfectly in accordance with the timeline in the accumulated analysis, since strong local relationships between adaptation rates and probability slopes during the 100–300 ms time windows resulted in an increase in the accumulated relationships from the beginning of the stimulus duration until the 350–400 ms time points. The analysis of the running window relationship for presentations 6–25 did not yield a significant correlation for any time bin. Taken together, these analyses demonstrate that the dynamics of adaptation were strongly related to the rapid improvements that we observe with repeated stimulus exposure.

#### Neural decoding accuracy is greater in the blocked than in the shuffled sequence

**Fig 6:**
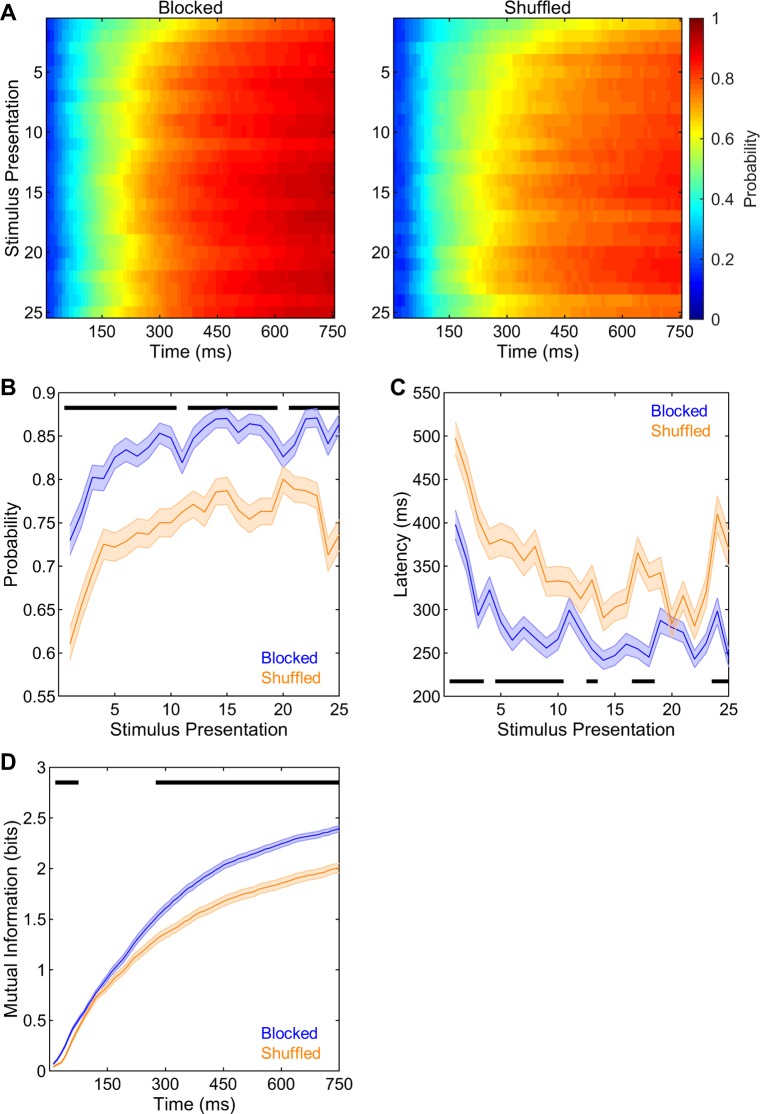
Neural decoding in blocked and shuffled sequences. (A) Neural decoding accuracies were analyzed across time points along the stimulus duration and stimulus presentations in blocked and shuffled sequences. (B) Correct neural decoding probabilities across stimulus presentations were higher in the blocked than in the shuffled sequence. (C) Correct neural decoding latencies across stimulus presentations were shorter in the blocked than in the shuffled sequence. (D) Mutual information was higher in the blocked than in the shuffled sequence between the 2^nd^ and 7^th^ time bins (20–70 ms) and from the 28^th^ time bin until the end of the stimulus period (280–750 ms). Shadings indicate SEMs. Horizontal black bars denote significant differences.

The above findings provide strong support for our main hypothesis that neural recognition of novel stimuli improves with repeated stimulus presentation. We next assessed the neural decoding differences between the blocked and shuffled stimulus presentation sequences. The correct neural decoding probabilities across time points along the stimulus duration and stimulus presentations are shown in **Fig 6A** for the blocked and the shuffled sequence. These figures were analyzed for probabilities and latencies separately as described above. The analysis of probabilities at different time points all produced similar findings, thus only the results at the 500 ms time point are presented here. Across stimulus presentations, correct neural decoding probabilities were dramatically higher in the blocked than in the shuffled sequence (F(1,334) = 35.39, p < 0.001, **Fig 6B**). There were also significant increases in correct neural decoding probabilities across stimulus presentations (F(24,8016) = 31.88, p < 0.001, **Fig 6B**). Furthermore, there was a significant interaction between sequence and stimulus presentation (F(24,8016) = 3.06, p < 0.001, **Fig 6B**); however, the detailed examination of this interaction via the analysis of the slopes of correct neural decoding probabilities for presentations 1–6 and 6–25 did not reveal either a sequence main effect (F(1,334) = 1.45, p = 0.229) or an interaction effect (F(1,334) = 1.38, p = 0.242). Overall, the slopes were significantly higher for presentations 1–6 than for presentations 6–25 (F(1,334) = 97.72, p < 0.001), indicating much more pronounced improvements in correct neural decoding probabilities during earlier than during later stimulus presentations. Taken together, these findings clearly show that neural decoding of stimulus identities is enhanced when stimuli are presented one-by-one in a blocked order as compared to when they are presented in a completely unpredictable, shuffled sequence. However, despite this overall difference, neural decoding accuracies improve at comparable rates with stimulus presentations between the two sequences.

The analysis of the correct neural decoding latencies for different probability levels revealed similar patterns, thus only the results for the 0.75 probability level are presented here. Overall, latencies were significantly shorter in the blocked than in the shuffled sequence (F(1,334) = 26.16, p < 0.001, **Fig 6C**). Moreover, there were significant changes in correct neural decoding latencies across stimulus presentations (F(24,8016) = 28.41, p < 0.001, **Fig 6C**). The interaction between stimulus presentation and sequence was also significant (F(24,8016) = 4.07, p < 0.001, **Fig 6C**); nevertheless, the analysis of the slopes of correct neural decoding latencies for presentations 1–6 and 6–25 did not yield a main effect of sequence (F(1,334) = 0.16, p = 0.689) or an interaction effect (F(1,334) = 0.49, p = 0.484). Overall, the slopes were significantly lower for presentations 1–6 than for presentations 6–25 (F(1,334) = 120.75, p < 0.001), showing stronger reductions in correct neural decoding latencies during early stimulus presentations. Taken together, these analyses show that the neural decoding of stimulus identities occurs much faster along the stimulus duration when stimuli are presented in a blocked sequence compared to when they are presented in shuffled order. Nevertheless, the changes in correct decoding latencies with stimulus presentation occurred at comparable rates between the two sequences.

The classifications resulting from the neural decoding process were also used to calculate the mutual information between true stimulus identities and neural response profiles as a function of the accumulated time along the stimulus duration. Across time bins, these mutual information estimations were significantly greater in the blocked than in the shuffled sequence (F(1,334) = 29.53, p < 0.001, **Fig 6D**). Not surprisingly, mutual information significantly increased with time (F(74,24716) = 2597.95, p < 0.001, **Fig 6D**). Most importantly, there was a significant interaction between sequence and time bins (F(74,24716) = 27.26, p < 0.001, **Fig 6D**). Planned comparisons indicated that mutual information was significantly greater in the blocked than in the shuffled sequence between the 2^nd^ and 7^th^ bins (20–70 ms) and again from the 28^th^ bin until the end of the stimulus duration (280–750 ms, all t(334) > 3.54, p < 0.05/75 for 75 separate time bins). Thus, the temporal profiles of neural responses are more informative about stimulus identities as early as 20 ms after the stimulus onset when stimuli are presented in a blocked order than when they are presented in a shuffled sequence.

#### More consistent neural responses to the same stimulus underlie more accurate neural decoding in the blocked than in the shuffled sequence

**Fig 7:**
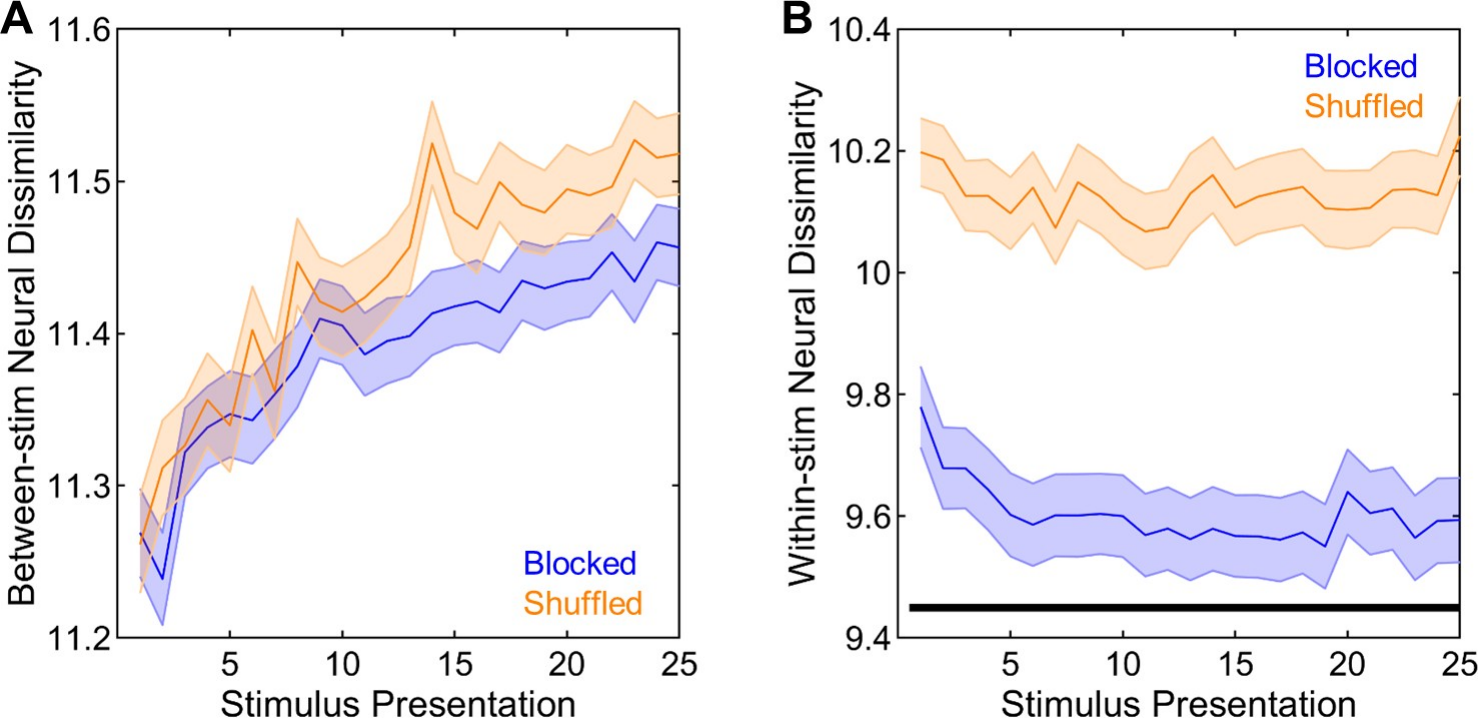
Neural dissimilarities in blocked and shuffled sequences. (A) Between-stimulus neural dissimilarities across stimulus presentations were comparable between the two sequences. (B) Within-stimulus neural dissimilarities in the blocked sequence were markedly lower than those in the shuffled sequence. Shadings indicate SEMs. Horizontal black bar denotes significant differences.

Next, between- and within-stimulus neural dissimilarities were compared between the two sequences to further probe the underlying dynamics that brought about the improved neural recognition in the blocked than in the shuffled sequence. Overall, between-stimulus neural dissimilarities were not significantly different between the blocked and the shuffled sequences (F(1,334) = 1.49, p = 0.223, **Fig 7A**). As described above, there were significant improvements in between-stimulus neural dissimilarities with stimulus presentation (F(24,8016) = 102.12, p < 0.001, **Fig 7A**). Furthermore, there was a significant interaction between stimulus presentation and sequence (F(24,8016) = 6.08, p < 0.001, **Fig 7A**); however, the detailed investigation of this interaction via the analysis of the slopes of between-stimulus neural dissimilarities for presentations 1–6 and 6–25 did not reveal either a sequence main effect (F(1,334) = 2.19, p = 0.140) or an interaction effect (F(1,334) = 0.19, p = 0.660). The slopes were significantly higher for presentations 1–6 than for presentations 6–25 (F(1,334) = 109.44, p < 0.001), indicating much more pronounced improvements in between-stimulus neural dissimilarities during earlier than during later stimulus presentations. Taken together, these analyses show that the dissimilarities between the neural responses to different stimuli, as well as the improvements in these dissimilarities with repeated stimulus exposure, were comparable between the blocked and shuffled sequences.

Within-stimulus neural dissimilarities, on the other hand, were dramatically higher in the shuffled sequence as compared to in the blocked sequence (F(1,334) = 32.60, p < 0.001, **Fig 7B**). In addition, within-stimulus neural dissimilarities showed significant changes with stimulus presentation (F(24,8016) = 19.86, p < 0.001. **Fig 7B**) and these changes were different between the blocked and shuffled sequences (F(24,8016) = 8.68, p < 0.001, **Fig 7B**). The analysis of the slopes of within-stimulus neural dissimilarities for presentations 1–6 and 6–25 revealed that the slopes were significantly lower for presentations 1–6 than for presentations 6–25 (F(1,334) = 152.92, p < 0.001) and in the blocked than in the shuffled sequence (F(1,334) = 25.11, p < 0.001). The interaction between stimulus presentation and sequence was also significant (F(1,334) = 16.64, p < 0.001); however, post-hoc comparisons showed that the slopes were significantly lower in the blocked than in the shuffled sequence for both presentations 1–6 and presentations 6–25 (both t(334) > 2.87, p < 0.005). As expected, the slopes were significantly lower for presentations 1–6 than for presentations 6–25 in both the blocked (t(183) = 11.04, p < 0.001) and the shuffled sequences (t(151) = 6.57, p < 0.001). The most crucial finding from these analyses is that the neural responses to any particular stimulus are much more consistent across presentations when stimuli are heard one-by-one in a blocked order than when they are heard in an unpredictable, shuffled sequence, and this is the main reason behind much more improved neural decoding in the blocked than in the shuffled sequence.

#### Narrow spike neurons are more informative about stimulus identities than wide spike neurons

**Fig 8:**
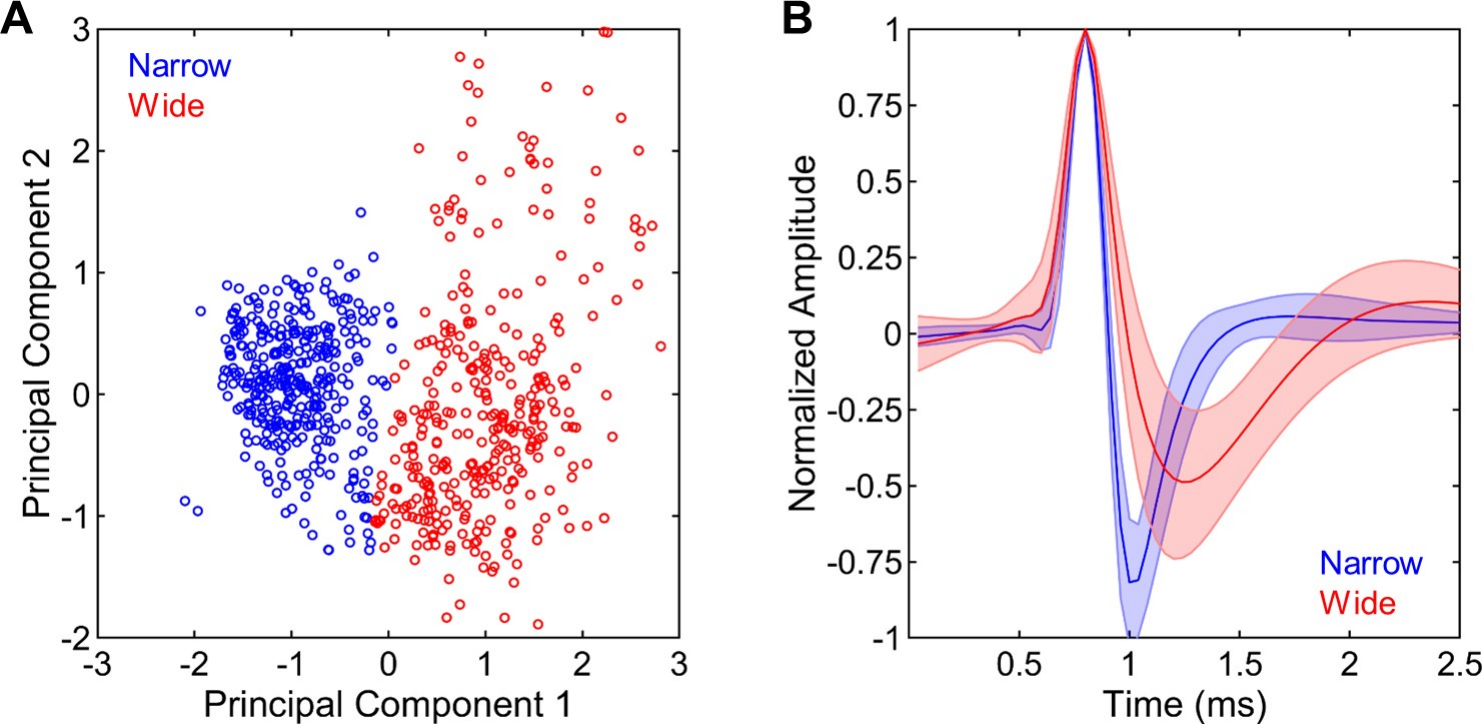
Categorization of single-units into narrow and wide spike neurons. (A) Single-units were divided into two clusters via affinity propagation based on the first two principal components underlying spike waveforms. (B) The resulting two clusters corresponded to neurons with wide and narrow spike waveforms.

**Fig 9:**
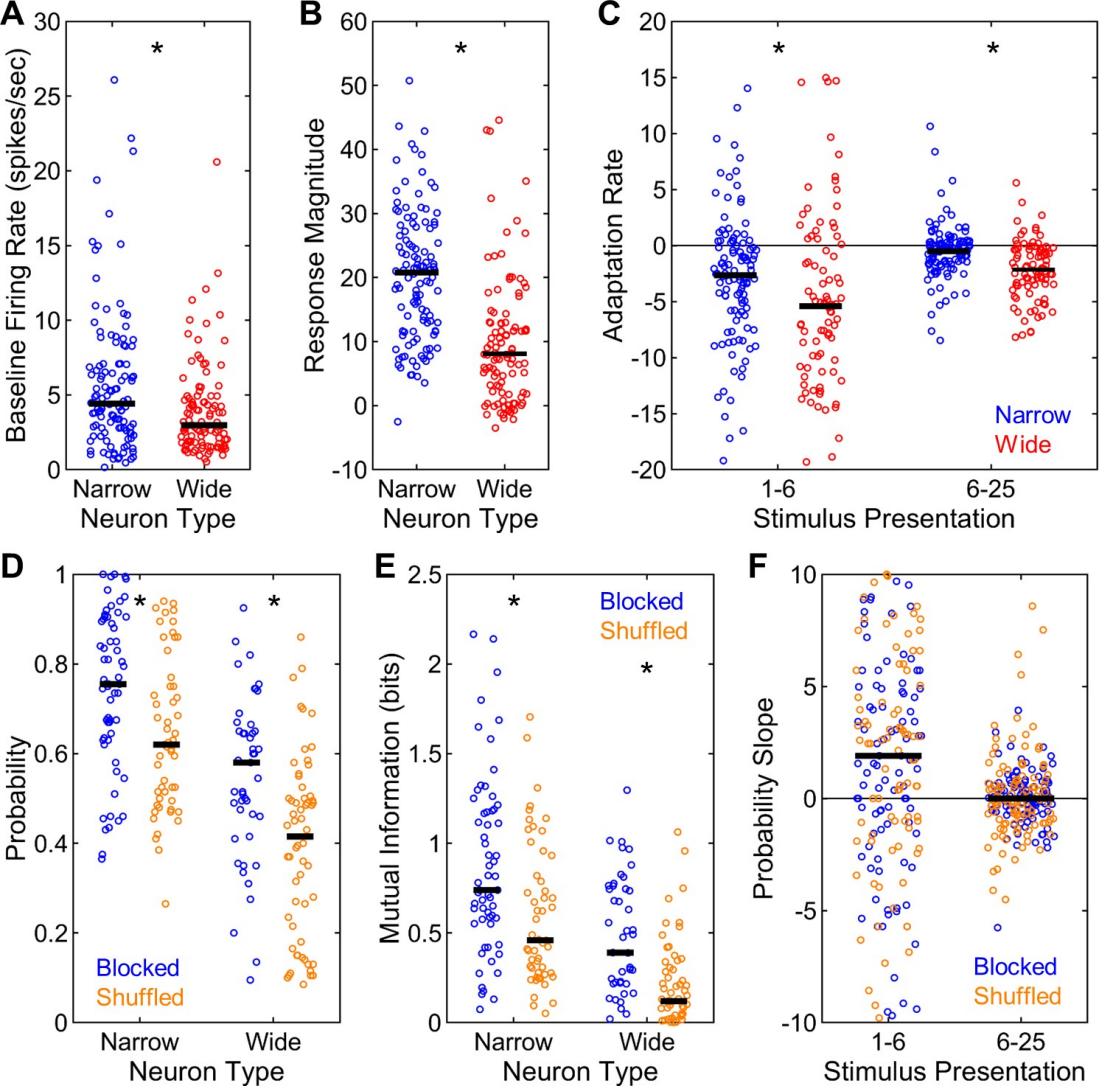
Single-unit response properties and neural decoding in blocked and shuffled sequences. (A) Firing rates during silent baseline conditions were higher for narrow than for wide spike neurons. (B) Stimulus-driven response magnitudes also showed the same effect. (C) Adaptation rates were more negative for wide then for narrow spike neurons both for presentations 1–6 and 6–25. (D) Correct neural decoding probabilities were higher in the blocked than in the shuffled sequence for both narrow and wide spike neurons. In addition, narrow spike neurons had higher correct neural decoding probabilities than did wide spike neurons in both the blocked and the shuffled sequence. (E) Mutual information also showed the same effects. (F) The slopes of correct neural decoding probabilities were higher than zero for presentations 1–6 than for presentations 6–25. Horizontal black bars indicate medians. Asterisks denote significant differences.

In addition to the analysis of multi-unit responses, single-unit spike trains were extracted by spike-sorting the raw neural recordings via an unsupervised technique [27]. The resulting single-units were classified into narrow and wide spike neurons based on their waveforms using an affinity propagation algorithm similar to previous reports [28] (**Fig 8**). This procedure classified 61 narrow and 45 wide spike neurons in the blocked sequence and 53 narrow and 60 wide spike neurons in the shuffled sequence. These distributions were not different between the neuron types or stimulus presentation sequences (X^2^(1) = 2.48, p = 0.115). Firing rates of narrow spike neurons during the silent baseline conditions were significantly greater than those of wide spike neurons (z = 2.92, p = 0.003, **Fig 9A**). Similarly, narrow spike neurons had markedly greater stimulus-driven response magnitudes compared to wide spike neurons (z = 7.40, p < 0.001, **Fig 9B**). Wide spike neurons, on the other hand, showed significantly more negative adaptation rates for both presentations 1–6 (z = 2.33, p = 0.020) and 6–25 (z = 4.72, p < 0.001, **Fig 9C**), indicating steeper adaptation. In sum, narrow spike neurons had higher baseline and stimulus-driven firing rates and displayed less adaptation with repeated stimulus presentation compared to wide spike neurons.

Next, the neural decoding differences were analyzed between the two neuron types as well as the two stimulus presentation sequences. Correct neural decoding probabilities were significantly greater for narrow than for wide spike neurons in both the blocked (z = 4.96, p < 0.001) and the shuffled sequence (z = 5.87, p < 0.001, **Fig 9D**). In addition, significantly greater probabilities were observed in the blocked than in the shuffled sequence for both narrow (z = 3.06, p = 0.002) and wide spike neurons (z = 3.83, p < 0.001, **Fig 9D**). The analysis of mutual information revealed exactly the same pattern of results. Mutual information estimations were significantly greater for narrow than for wide spike neurons in both the blocked (z = 4.80, p < 0.001) and the shuffled sequence (z = 5.59, p < 0.001, **Fig 9E**). Similarly, mutual information in the blocked sequence was significantly greater than those in the shuffled sequence both for narrow (z = 3.15, p = 0.002) and wide spike neurons (z = 3.79, p < 0.001, **Fig 9E**). Thus, the response profiles of narrow spike neurons are much more informative about stimulus identities compared to wide spike neurons. In addition, the more accurate stimulus decoding in the blocked than in the shuffled sequence observed with the multi-unit responses was exactly paralleled in the response profiles of single neurons.

Finally, the changes in neural decoding accuracy of single neurons as a function of stimulus presentation were examined via the analysis of the slopes of correct neural decoding probabilities for presentations 1–6 and 6–25. The slopes for either presentations 1–6 or 6–25 were not different between the neuron types or the sequences (all z < 1.01, p > 0.319). Altogether, the slopes of correct decoding probabilities were significantly greater than 0 for presentations 1–6 (z = 3.31, p = 0.001, **Fig 9F**), but not for presentations 6–25 (z = 0.43, p = 0.664). Thus, in parallel with the findings in multi-unit responses, the decoding of stimulus identities using single-unit neural responses also rapidly improves with initial presentations of novel acoustic signals.

### Experiment 2

Having confirmed the rapid improvements in neural recognition of novel stimuli with repeated stimulus presentation, we next tested whether these improvements are maintained over the long term, 20 hours after their initial induction. Sixteen male zebra finches were presented with 25 repetitions of each of 8 conspecific songs during awake, restrained electrophysiological recordings similar to Experiment 1. However, this time, half of the birds were pre-exposed to the test stimuli 20 hours before the electrophysiological recordings, while the other half was passively presented with other stimuli that were unrelated to the test songs. Only shuffled presentation sequences were used for both the pre-exposure and the test phase. There were 145 multi-unit sites in the pre-exposed group and 149 multi-unit sites in the control group histologically verified to be in NCM. In addition, 52 single-units in the pre-exposed and 63 single-units in the control condition were sorted from these multi-unit recordings. Preliminary analyses did not show systematic lateral differences, thus the two hemispheres were combined for all subsequent analyses. All analyses were based on multi-unit responses, unless specified as single-units.

#### Adaptation occurs faster in the pre-exposed than in the control condition

**Fig 10:**
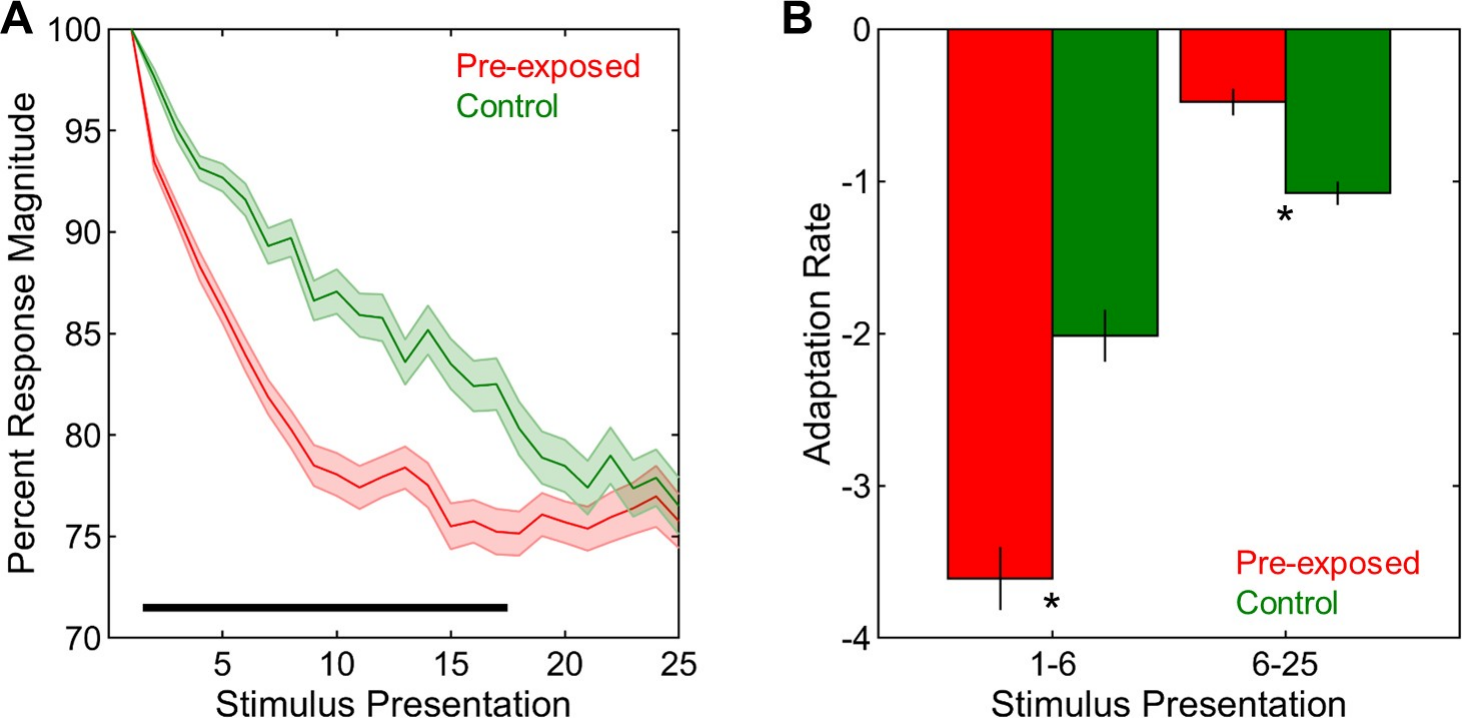
Adaptation in pre-exposed and control conditions. (A) Percent response magnitudes across stimulus presentations were smaller in the pre-exposed than in the control condition. (B) Adaptation rates in the pre-exposed condition were more negative for presentations 1–6 and less negative for presentations 6–25 than those in the control condition. Shadings and error bars indicate SEMs. Horizontal black bar and asterisks denote significant differences.

First, the presence of a neural memory for previously familiarized stimuli in the pre-exposed condition was assessed via the analyses of response magnitudes and adaptation rates, similar to previous studies [17,26]. Across stimulus presentations, percent response magnitudes were significantly greater in the control than in the pre-exposed condition (F(1,292) = 16.68, p < 0.001, **Fig 10A**). There were also significant changes in the percent response magnitudes with stimulus presentation (F(23,6716) = 225.35, p < 0.001, **Fig 10A**). Critically, these changes were different in the two exposure conditions (F(23,6716) = 13.81, p < 0.001, **Fig 10A**). To further examine these differences in detail, the adaptation rates for presentations 1–6 and 6–25 were analyzed separately. Overall, adaptation rates were more negative in the pre-exposed than in the control exposure condition (F(1,292) = 10.41, p < 0.001, **Fig 10B**) and also for presentations 1–6 than for presentations 6–25 (F(1,292) = 223.28, p < 0.001, **Fig 10B**). Most importantly, there was a strong interaction between exposure and stimulus presentation (F(1,292) = 64.90, p < 0.001, **Fig 10B**), such that, for presentations 1–6, adaptation rates were significantly more negative in the pre-exposed than in the control condition (t(292) = 5.97, p < 0.001), whereas this pattern was completely reversed for presentations 6–25, indicating significantly more negative adaptation rates in the control than in the pre-exposed condition (t(292) = 5.16, p < 0.001). Looking at the interaction from the other perspective, adaptation rates for presentations 1–6 were significantly more negative compared to those for presentations 6–25 in both the pre-exposed (t(144) = 13.82, p < 0.001) and the control exposure condition (t(148) = 6.11, p < 0.001). Taken together, these analyses clearly demonstrated a neural memory for the test songs in birds that were passively exposed to those stimuli 20 hours earlier. Neural responses adapted more rapidly and remained at asymptotic levels in pre-exposed birds, whereas the typical gradual adaptation profile for novel signals was observed in control birds that were hearing the test stimuli for the first time in life.

#### Neural decoding accuracy is improved in the pre-exposed compared to the control condition

**Fig 11:**
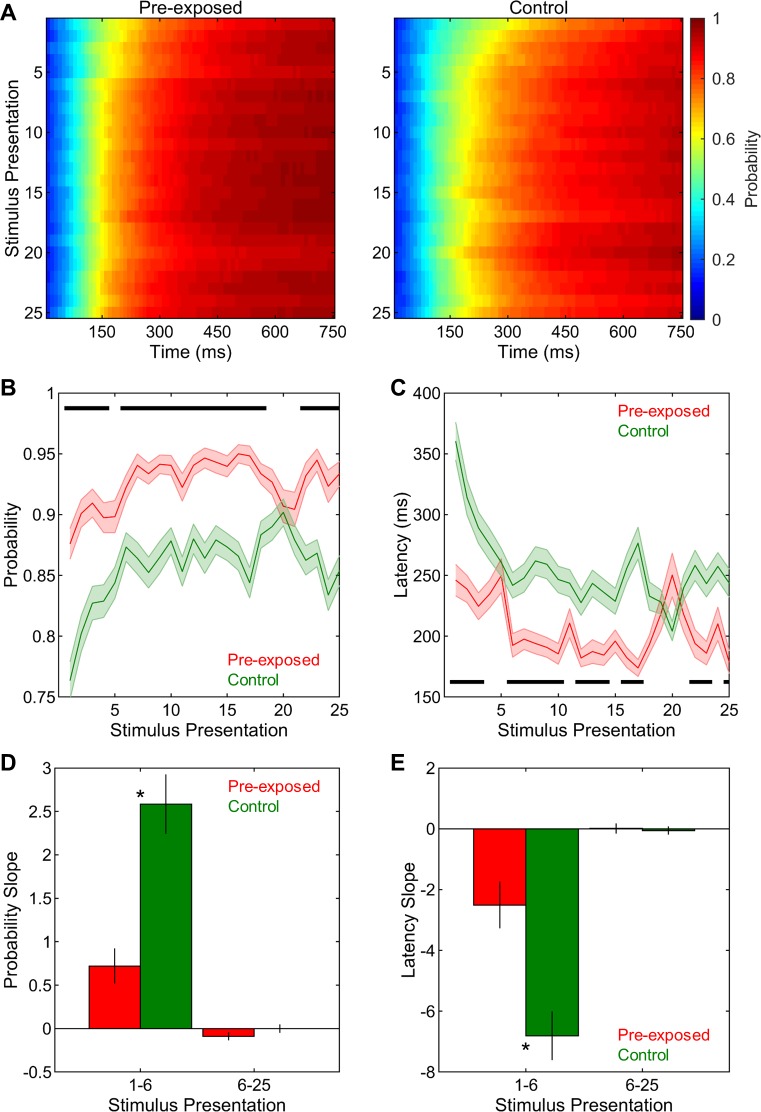
Neural decoding in pre-exposed and control conditions. (A) Neural decoding accuracies were analyzed across time points along the stimulus duration and stimulus presentations in pre-exposed and control conditions. (B) Correct neural decoding probabilities across stimulus presentations were higher in the pre-exposed than in the control condition. (C) The slopes of correct neural decoding probabilities were lower in the pre-exposed than in the control conditions for presentations 1–6. (D) Correct neural decoding latencies across stimulus presentations were shorter in the pre-exposed than in the control condition. (E) The slopes of correct neural decoding latencies were less negative in the pre-exposed than in the control conditions for presentations 1–6. Shadings and error bars indicate SEMs. Horizontal black bars and asterisks denote significant differences.

Having shown the presence of a neural memory for previously familiarized stimuli in the pre-exposed condition, we next assessed whether the decoding of stimulus identities using the temporal profiles of neural responses was also improved when those stimuli had been heard 20 hours earlier compared to when they were completely novel. The correct neural decoding probabilities across time points along the stimulus duration and stimulus presentations are shown in **Fig 11A** for the pre-exposed and control exposure conditions. Overall, correct neural decoding probabilities at the 500 ms time point were significantly greater in the pre-exposed than in the control condition (F(1,292) = 35.20, p < 0.001, **Fig 11B**). There were significant changes in probabilities with stimulus presentation (F(24,7008) = 14.77, p < 0.001, **Fig 11B**) and these changes were different between the pre-exposed and control conditions (F(24,7008) = 4.28, p < 0.001, **Fig 11B**). Thus, these differences were further examined via the analysis of the slopes of probabilities for presentations 1–6 and 6–25. Generally, the slopes of probabilities were significantly greater in the control than in the pre-exposed group (F(1,292) = 23.24, p < 0.001, **Fig 11D**) and for presentations 1–6 than for presentations 6–25 (F(1,292) = 70.15, p < 0.001, **Fig 11D**). Most importantly, exposure and stimulus presentation interacted strongly (F(1,292) = 19.16, p < 0.001, **Fig 11D**). For presentations 1–6, the probabilities in the control condition had significantly greater slopes than did those in the pre-exposed condition (t(292) = 4.67, p < 0.001), whereas there was no difference between the two exposure conditions for presentations 6–25 (t(292) = 1.32, p = 0.188). The slopes of probabilities were similarly greater for presentations 1–6 than for presentations 6–25 in both the pre-exposed (t(144) = 4.22, p < 0.001) and the control condition (t(148) = 7.32, p < 0.001). Taken together, these findings strongly support our hypothesis that the rapid gains in neural decoding with repeated stimulus exposure lasts for at least 20 hours as indicated by better neural decoding performance for the same stimuli when they are previously familiarized as compared to when they are heard for the first time in life. As a result of already high neural decoding accuracy in the pre-exposed condition from the beginning of the stimulus presentation, rapid improvements are much more pronounced when the stimuli are novel as compared to when they are familiar.

The latencies along the stimulus duration to reach the probability level of 0.750 ms were also analyzed across stimulus presentation in the two exposure conditions. Correct neural decoding latencies were generally shorter in the pre-exposed than in the control condition (F(1,292) = 21.78, p < 0.001, **Fig 11C**). Latencies also changed significantly with stimulus presentation (F(24,7008) = 14.80, p < 0.001, **Fig 11C**). These changes different significantly between the two conditions (F(24,7008) = 6.92, p < 0.001, **Fig 11C**) and thus were investigated further via the analysis of the slopes of latencies for presentations 1–6 and 6–25 separately. Overall, these slopes were significantly more negative in the control than in pre-exposed condition (F(1,292) = 15.18, p < 0.001, **Fig 11E**) and also for presentations 1–6 than for presentations 6–25 (F(1,292) = 65.81, p < 0.001, **Fig 11E**). Critically, there was a significant interaction between exposure and stimulus presentation (F(1,292) = 13.72, p < 0.001, **Fig 11E**), such that, for presentations 1–6, the slopes of latencies were significantly more negative in the control than in the pre-exposed condition (t(292) = 3.87, p < 0.001), while no such difference was observed between the two exposure conditions for presentations 6–25 (t(292) = 0.31, p = 0.757). The slopes of latencies were significantly more negative for presentations 1–6 than for presentations 6–25 in both the pre-exposed (t(144) = 3.21, p = 0.002) and the control condition (t(148) = 8.14, p < 0.001). In sum, the long-term effects of passive stimulus exposure on neural decoding accuracies are paralleled in latencies, showing earlier correct neural decoding latencies for the same stimuli when they are previously familiarized as compared to when they are not. Again, due to already low levels of neural decoding latencies in the pre-exposed condition, rapid changes in latencies are more pronounced when the stimuli are novel as compared to when they are heard 20 hours earlier.

**Fig 12:**
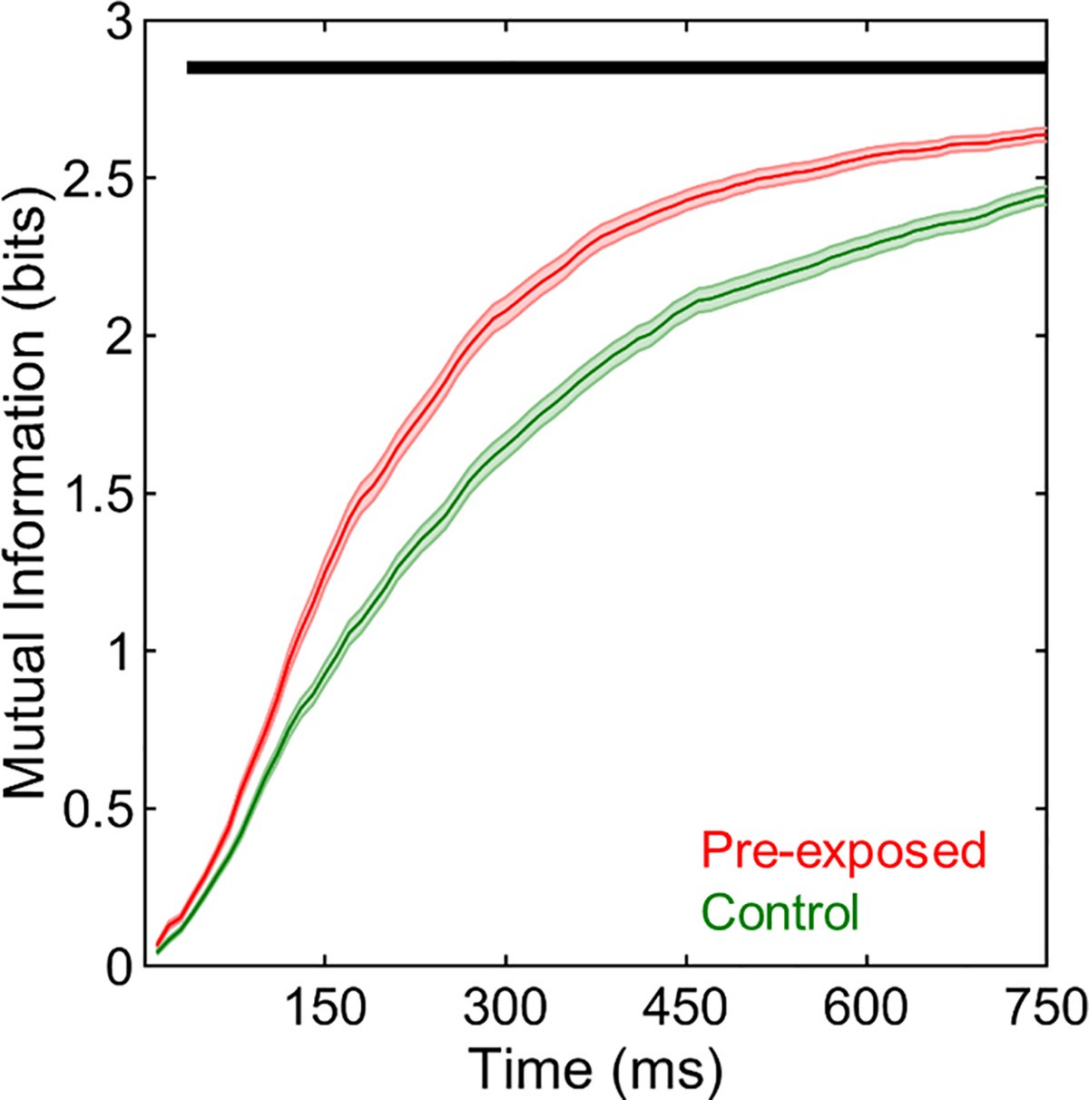
Mutual information in pre-exposed and control conditions. Mutual information was higher in the pre-exposed than in the control condition starting from the 4^th^ time bin until the end of the stimulus period (40–750 ms). Shadings indicate SEMs. Horizontal black bar denotes significant differences.

Finally, mutual information between stimulus identities and neural response profiles were also compared between the pre-exposed and control conditions. Generally, mutual information was significantly greater in the pre-exposed than in the control exposure condition (F(1,292) = 50.00, p < 0.001, **Fig 12**). Not surprisingly, mutual information estimations significantly increased across time bins (F(74,21608) = 3993.78, p < 0.001, **Fig 12**). Furthermore, there was a significant interaction between sequence and time bin (F(74,21608) = 22.03, p < 0.001, **Fig 12**). Further planned comparisons indicated that mutual information was significantly greater in the pre-exposed than in the control exposure condition starting from the 4^th^ bin until the end of the stimulus period (40–750 ms, all t(292) > 3.64, p < 0.05/75 for 75 separate time bins). Thus, the temporal profiles of neural responses are more informative about the stimulus identities as early as 40 ms after the stimulus onset when stimuli are heard 20 hours earlier as compared to when they are completely novel.

#### Neither narrow nor wide spike neurons show improved neural decoding accuracy in the pre-exposed compared to the control condition

**Fig 13:**
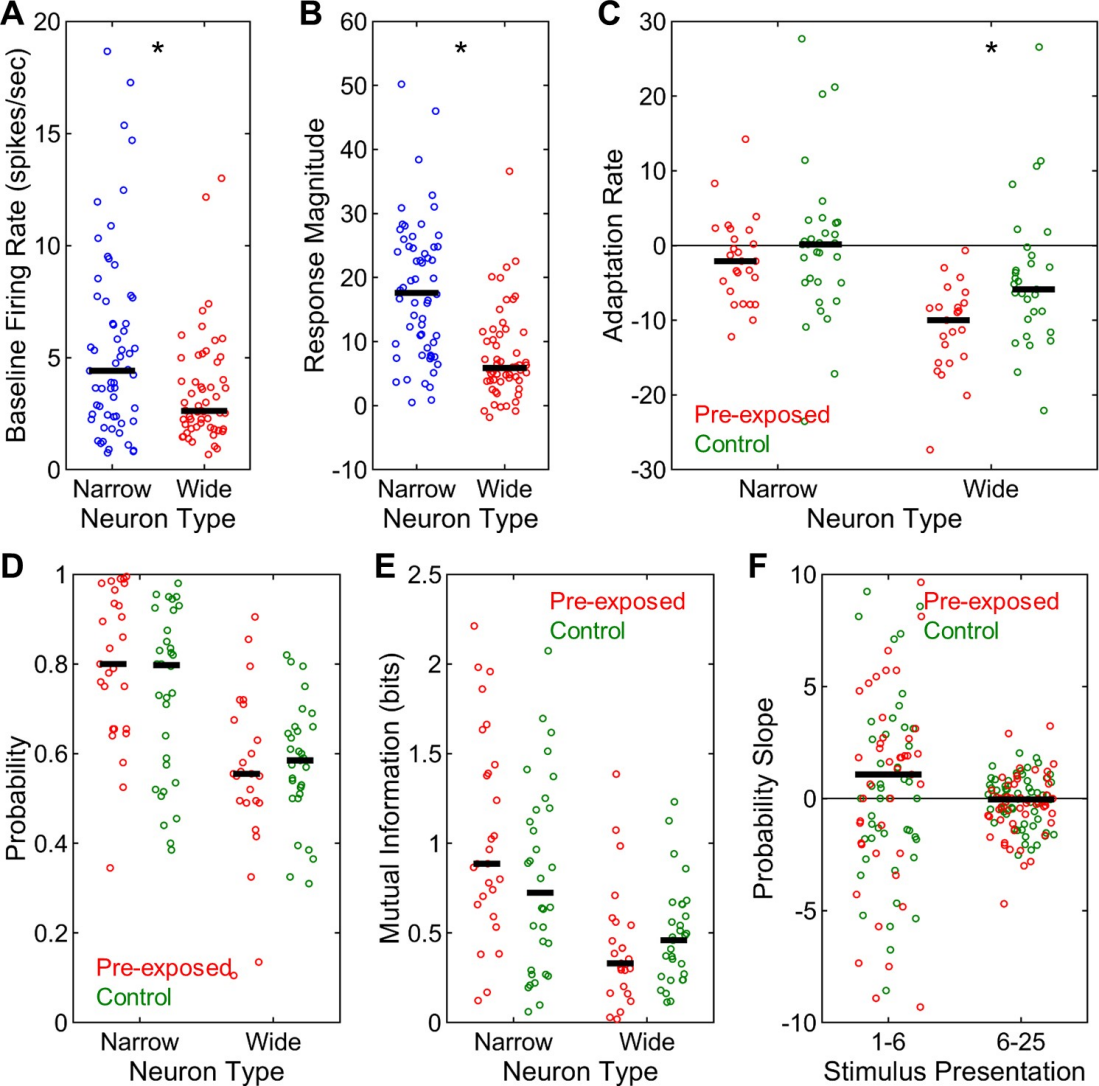
Single-unit response properties and neural decoding in pre-exposed and control conditions. (A) Firing rates during silent baseline conditions were higher for narrow than for wide spike neurons. (B) Stimulus-driven response magnitudes also showed the same effect. (C) Adaptation rates for presentations 1–6 were more negative in the pre-exposed than in the control condition for wide, but not for narrow, spike neurons. (D) Narrow spike neurons had higher correct neural decoding probabilities than did wide spike neurons in both the pre-exposed and the control condition. However, correct neural decoding probabilities were not different between the two conditions either for narrow or for wide spike neurons. (E) Mutual information also showed the same effects. (F) The slopes of correct neural decoding probabilities were higher than zero for presentations 1–6, but not for presentations 6–25. Horizontal black bars indicate medians. Asterisks denote significant differences.

There were 27 narrow and 25 wide spike neurons in the pre-exposed condition and 32 narrow and 31 wide spike neurons in the control condition. These distributions were not different between the neuron types or the exposure conditions (X^2^(1) = 0.08, p = 0.772). Exactly as in Experiment 1, narrow spike neurons displayed significantly higher baseline firing rates (z = 2.65, p = 0.008, **Fig 13A**) as well as stimulus-driven response magnitudes (z = 5.53, p < 0.001, **Fig 13B**) as compared to wide spike neurons. In contrast, wide spike neurons had significantly more negative adaptation rates than did narrow spike neurons for both presentations 1–6 (z = 4.45, p < 0.001) and 6–25 (z = 3.62, p < 0.001, **Fig 13C**). We also tested whether there was a neuronal memory for previously heard signals via the comparison of adaptation rates between the two exposure conditions for presentations 1–6 and 6–25 similar to the analysis of multi-unit responses. For narrow spike neurons, adaptation rates were not significantly different between the pre-exposed and control conditions either for presentations 1–6 (z = 1.05, p = 0.294) or 6–25 (z = 0.20, p = 0.839). Wide spike neurons, on the other hand, had significantly more negative slopes in the pre-exposed compared to the control condition for presentations 1–6 (z = 2.55, p = 0.011, **Fig 13C**), whereas, no such difference was observed for presentations 6–25 (z = 0.22, p = 0.827). Thus, during initial stimulus presentations, adaptation was faster when those stimuli were previously familiarized than when they were completely novel for wide, but not for narrow, spike neurons. This suggests that neural memory for previously heard signals might be primarily coded by wide spike neurons.

The correct neural decoding probabilities were significantly greater for narrow than for wide spike neurons in both the pre-exposed (z = 4.30, p < 0.001, **Fig 13D**) and the control condition (z = 3.25, p = 0.001, **Fig 13D**). However, contrary to our predictions, there was no significant difference in probabilities between the two exposure conditions either for narrow (z = 1.50, p = 0.134) or for wide spike neurons (z = 0.56, p = 0.575). In a similar vein, mutual information estimations did not significantly differ between the pre-exposed and control conditions either for narrow (z = 1.66, p = 0.097) or for wide spike neurons (z = 0.72, p = 0.473), although narrow spike neurons had significantly higher mutual information than did wide spike neurons in both the pre-exposed (z = 4.39, p < 0.001, **Fig 13E**) and the control condition (z = 2.87, p = 0.004, **Fig 13E**). Thus, unlike for multi-unit responses, neural decoding accuracies were not different in single-units when the same stimuli were passively familiarized 20 hours earlier or when they were completely novel.

Finally, the changes in neural decoding accuracy of single neurons as a function of stimulus presentation were examined via the analysis of the slopes of correct neural decoding probabilities for presentations 1–6 and 6–25 separately. The slopes for either presentations 1–6 or 6–25 were not different between the neuron types or exposure conditions (all z < 1.41, p > 0.161). Together, the slopes of correct decoding probabilities were significantly greater than 0 for presentations 1–6 (z = 2.11, p = 0.035, **Fig 13F**), whereas no such difference was observed for presentations 6–25 (z = 1.35, p = 0.177). Thus, as for multi-unit responses, neural decoding accuracies for single-unit responses rapidly improve with few initial stimulus presentations.

## Discussion

We investigated the effects of passive exposure on neural recognition of novel natural vocalizations in zebra finch NCM in two experiments. Experiment 1 provided strong evidence that the temporal profiles of neural responses to different novel signals rapidly become more dissimilar from each other with repeated exposure, which improves the decoding of these stimuli from neural responses. These rapid improvements in neural decoding accuracies were tightly related to the process of adaptation in NCM. In addition, the results of Experiment 1 indicated that the temporal profiles of neural responses are sensitive to the sequence in which the signals are presented, such that neural responses are more informative about acoustic stimuli when they are presented in a blocked than in a shuffled sequence. Experiment 2 supported and extended these findings by showing that the rapid gains in neural decoding of natural vocalizations with passive familiarization remained in effect 20 hours after the initial encounter. Finally, the results of both experiments demonstrated that the activity of NCM neurons with narrow spike waveforms was more informative about stimulus identities compared to the responses of wide spike neurons.

### Rapid improvements in neural decoding accuracy

Neural decoding of novel acoustic signals using the temporal profiles of neural responses dramatically improved with passive stimulus exposure. The results of Experiment 1 clearly showed that these improvements in neural recognition were very rapid, exhibiting a sharp increase during initial stimulus presentations and either little or no change with further exposure. These improvements with passive exposure were paralleled by a reduction in the latency along the stimulus duration to accurately decode stimulus identities. This means that, as they become more familiar, novel signals can be recognized by hearing shorter sections from the beginning of the stimulus. Rapid improvements in neural decoding accuracies observed in multi-unit responses were paralleled in single-unit responses, which suggests that the changes in the temporal profiles of responses observed in the neural population most likely result from modifications in spike timing of individual neurons in response to repeated stimulus presentation. Furthermore, the results of Experiment 2 replicated these findings, revealing rapid improvements in neural decoding accuracies and reductions in neural decoding latencies exactly as in Experiment 1. Taken together, these findings strongly show that passive familiarization with novel acoustic signals through repeated exposure rapidly and dramatically improves their neural recognition. These results are in line with a very recent study describing an adaptation process with similar long-term dynamics as seen in the songbird NCM in the secondary, but not in the primary, auditory cortex of ferrets [19]. Mutual information between stimulus identities and neural responses were shown to increase from the first 25 to the immediately following second 25 repetitions of the same complex signals in the secondary auditory cortex. Our findings not only corroborate these effects, but also extend them by showing the rapid trial-by-trial dynamics and the long-term characteristics of neural decoding improvements with passive exposure in the songbird higher-order auditory nucleus NCM.

Detailed analysis of the dynamics underlying these rapid improvements in neural decoding accuracies showed that between-stimulus neural dissimilarities increased, while within-stimulus neural dissimilarities decreased during the initial stimulus presentations. This means that the temporal profiles of neural responses to any given stimulus became more consistent for that stimulus and more differentiated from the temporal profiles of neural responses to other stimuli. These dynamics together resulted in the enhancement of neural decoding accuracies during these early stimulus presentations. During the following stimulus repetitions, however, robust improvements in between-stimulus neural dissimilarities co-occurred with slight increases in within-stimulus neural dissimilarities. These changes in within-stimulus neural dissimilarities were unexpected and suggested that the reliability of neural representations marginally dropped with further exposure to the same signals. As the net effect of these two changes during later stimulus presentations, gains in neural decoding accuracy either continued at a much more modest rate or were completely absent.

### The relationship between adaptation and neural decoding accuracy improvements

The main working hypothesis explored in this study was that the rapid improvements in neural recognition of acoustic signals induced by passive exposure were related to the process of adaptation in NCM. In Experiment 1, the magnitude of neural responses adapted with repeated exposure to the same signals, as expected from the well-known phenomenon of adaptation in NCM [8–10]. We analyzed the relationship between the rates of adaptation and the rates of improvements in neural decoding accuracies in detail. As hypothesized, the recording sites that underwent stronger adaptation also showed more robust gains in neural decoding accuracies during initial stimulus presentations. This relationship was strongest when the first ~400 ms of the neural response from the stimulus onset was taken into account for neural decoding. Interestingly, the strength of this relationship dropped when the time points later than the first 400 ms were also included in neural decoding, which suggests that the process of adaptation in these later time points did not help to improve neural decoding accuracies. These findings were further corroborated by the analysis of the same relationship conducted separately for different time windows along the stimulus duration. The relationship between adaptation rates and neural decoding improvements were strongest in the time windows from 100 to 300 ms along the stimulus duration, whereas adaptation after the 300 ms generally did not relate to neural decoding improvements. During the most optimal time windows, rates of adaptation accounted for 14% of the variation in the rates of neural decoding improvements.

It is essential to note that the reported relationship between the process of adaptation and the improvements in neural decoding accuracies in NCM cannot be artifactual. The neural decoding algorithm used throughout this study involved a normalization step to control for the changes in total response magnitudes of neural activity across stimulus presentations. Thus, neural decoding was only sensitive to the changes in the temporal profiles of neural responses across stimulus presentations. The process of adaptation, on the other hand, was calculated by taking into account only the changes in total response magnitudes, and completely disregarding the changes in the temporal patterns of activity, across stimulus presentations. Thus, this neural decoding algorithm was specifically developed to control for any portion of the correlation that might be artifactual in the analysis of the relationship between adaptation rates and neural decoding accuracy improvements. As a result, we believe that even the strongest relationships reported in the present study likely represent lower bounds and that much more robust relationships would be observed if the raw responses were taken into account for neural decoding without any normalization.

The relationship between adaptation and neural decoding improvements most likely indicates that these phenomena are the two different manifestations of the same underlying mechanism in NCM. The phenomenon of adaptation to features or complex statistics of external signals studied in the mammalian brain does not necessarily imply that the neural response magnitudes decrease with repeated exposure. Rather, the receptive fields of neurons change in complex ways to adjust the dynamic range of neurons to the relevant stimulus statistics in the environment [29,30]. In a similar vein, recent studies in our lab demonstrated that the spectro-temporal receptive fields of neurons in NCM undergo rapid changes with repeated stimulus exposure (Yang & Vicario, unpublished results). In fact, adaptation may just be a measure of dynamic changes in receptive field selectivity, with more selective receptive fields showing smaller responses with repeated stimulation. If this formulation is correct, this improved selectivity in the receptive fields would also lead to more stimulus-selective responses, increasing the accuracy of the neural decoding of stimulus identities with stimulus repetition.

### Stimulus sequence effects on neural decoding accuracy

In Experiment 1, neural decoding of the same novel stimuli was more accurate and occurred faster when those signals were presented in a blocked compared to in a shuffled sequence. Mutual information between the temporal profiles of neural responses and stimulus identities also showed the same effect. This effect was clear in both multi-unit and single-unit responses, suggesting that the stimulus presentation sequence modulated temporal profiles of neural activity at the individual neuron level. This difference between the two stimulus presentation sequences was due to the differences in between- and within-stimulus neural dissimilarities. The dissimilarities between the temporal profiles of neural responses to different stimuli were generally similar between the two stimulus presentation sequences. However, the within-stimulus dissimilarities for any particular stimulus were strikingly higher in the shuffled than in the blocked sequence, which suggests that the temporal profiles of neural responses to repeated presentations of a particular stimulus were much less consistent in the shuffled sequence. The net effect of these two opposing factors was that the ability to identify acoustic signals using the temporal profiles of neural responses was diminished in the shuffled compared to the blocked sequence. Taken together, these findings suggest that the more predictable context produced by the blocked stimulus presentation sequence, in contrast to the unpredictable random stimulus transitions in the shuffled sequence, led to more reliable neural response profiles across repetitions of any given stimulus, which in turn enhanced the neural differentiation among different stimuli.

Similar findings were reported in studies that investigated the effects of talker variability on speech comprehension. Recognition of speech signals is poorer and takes more time when stimuli are spoken by different talkers in a shuffled setting as compared to when utterances from different speakers are presented one by one [31,32]. These findings have been interpreted as an indication of a talker normalization process that more successfully improves the mapping of sounds to phonetic categories by accumulating talker-specific evidence under the stable conditions of a blocked setup as compared to a shuffled sequence, which triggers the normalization process every time the talker changes and leads to discontinuities in incoming talker-specific vocalization characteristics. Although in the case of zebra finch vocalizations it is impossible to hypothesize a mapping mechanism between sounds and phoneme-like abstract processing units, either due to the absence of, or our ignorance of, such a system, the difference between blocked and shuffled sequences seems to reflect similar principles. The main drive behind the neural decoding accuracy and mutual information differences was that the temporal profiles of neural responses to different presentations of any particular signal were much more consistent in the blocked than in the shuffled presentation. Thus, similar to the proposed talker normalization process in humans, the discontinuities in the presentations of any particular stimulus in the shuffled sequence may induce variability and thus hinder the modulation of neural response profiles to reliably represent acoustic signals in NCM. Interestingly, the well-known intermixed-blocked effect in perceptual learning indicates that stimulus discrimination is enhanced following an intermixed (shuffled) passive pre-exposure compared to a blocked one [33]. This effect has been primarily investigated in the visual domain [34–36], but it is also observed in flavor discrimination [37]. At first sight, this phenomenon might seem contradicting with the present results. However, the critical difference between the two sets of findings is that our study shows enhanced discrimination for the blocked presentation during the passive exposure itself, while the intermixed-blocked effect shows the diminished performance for the blocked presentation during a subsequent test performance. The highly similar contextual interference effect seen in verbal [38] and motor learning [39,40] also indicates a poorer performance for blocked training in retrieval tests. Nevertheless, the counterintuitive other side of the contextual interference effect is that, despite the poorer subsequent test performance, blocked training leads to more enhanced initial practice performance compared to intermixed training. This initial enhanced performance for blocked presentation is perfectly in line with the present findings. For the subsequent test performance, we have only used shuffled presentation during the initial pre-exposure in Experiment 2. Thus, we do not know whether the blocked pre-exposure would lead to less accurate stimulus discrimination in the 20-hour memory test compared to the shuffled pre-exposure, in line with the contextual interference effect. Taken together, our experimental approach and analytical tools in zebra finches enable the in-depth investigation of the neural basis of these well-known effects seen at the behavioral level in humans.

### Long-lasting improvements in neural decoding accuracy

As shown in previous studies, the results of Experiment 2 demonstrated that neural responses to novel stimuli in NCM gradually adapted with repeated exposure, whereas the responses to signals that were passively familiarized 20 hours earlier decreased dramatically with few repetitions and remained at adapted levels thereafter. This marked difference between the adaptation profiles of novel and familiar signals is taken as an indication of neural auditory memory in NCM [17,26]. It is important to note that the initially high levels of responding decreasing dramatically to the previously adapted levels for the pre-exposed signals with few repetitions seem to indicate a two-process explanation for adaptation in NCM. The first, which might be called a detection process, shows an initial high responding during the first few presentations of a signal within a playback episode, whether the stimulus is completely novel or has been familiarized before. The second, which might be called an encoding process, indicates more moderate levels of responding, which shows gradual adaptation, during the subsequent stimulus repetitions. Taken into account these two processes together, both the pre-exposed and novel signals elicit similarly strong responses during the initial detection process. However, during the following encoding process, responding to the previously encoded signals quickly decreases to the already adapted levels, while the novel signals undergo more gradual adaptation for their encoding of the first time. This results in a steeper slope in the pre-exposed than in the control condition during the first few stimulus presentations. In contrast, during following stimulus presentations, we see steeper adaptation for the control compared to the pre-exposed condition, because the previously encoded stimuli remain at the already adapted levels and show little further adaptation, whereas novel signals continue their gradual adaptation for further encoding. These adaptation dynamics were first reported by Chew et al. [9] and exactly replicated in the present study.

The differences in adaptation profiles were paralleled by higher levels of neural decoding accuracy and mutual information for exactly the same stimuli when they had been previously familiarized as compared to when they were heard for the first time. Furthermore, a given level of neural decoding accuracy was reached sooner along the stimulus duration in the pre-exposed than in the control condition. Taken together, there seems to be a link between conditions that produce long-term adaptation and neural decoding improvements. However, a closer examination of the trial-by-trial dynamics reveals interesting details. For instance, it is well-established that presentation of a novel song for 20 times does not produce a long-term neural memory that can be detected 20 hours later in NCM [9,10]. In Experiment 2, 200 stimulus repetitions were used for passive familiarization, which successfully induced long-term memory. However, the results of Experiments 1 and 2 indicate that there is little or no gain in neural decoding accuracy after about the first 6 stimulus presentations. This suggests that adaptation during the later stimulus presentations does not contribute to rapid improvements in neural decoding, but is needed for consolidation of those improvements for later processing. The direct relationships between rapid and long-term effects are hard to decipher from the experiments in this study, because electrophysiological responses from given sites and neurons were recorded either only during the test phase or only during the initial induction phase of memories. That is, there was no longitudinal recording. Having now established the first clear evidence, to our knowledge, for long-lasting gains in neural decoding with passive exposure, future studies utilizing chronic recordings are needed to address the dynamics governing the amount of information retained or lost at particular sites after the initial familiarization of novel acoustic signals.

Contrary to findings in multi-unit responses, single-unit responses in Experiment 2 did not show all these effects. To begin with, adaptation rates were different when the same stimuli were previously heard as opposed to when they were completely novel only during the first 6 stimulus presentations and for wide spike neurons, whereas no difference was observed for narrow spike neurons. This suggests that the long-term neural memory for previously familiarized acoustic signals might be coded by wide, but not narrow, spike neurons in NCM. Regardless of this difference, neither neural decoding accuracies nor the mutual information between neural responses and stimulus identities differed between the pre-exposed and control exposure conditions for single neurons, unlike in multi-unit responses. Whether this difference between the multi-unit and single-unit findings points to a fundamental difference in encoding of the long-term improvements in neural recognition of acoustic signals at the single neuron versus the neural population level or to methodological limitations in the present study is not clear. The latter possibility appears more likely because these findings were based on a limited number of single-units (n = 52 for pre-exposed; n = 63 for control). The yield of single-units was substantially lower in Experiment 2 than in Experiment 1, for reasons that are not understood. As a result, we believe that our findings concerning long-term improvements in neural decoding in single neurons are provisional and thus call out for an examination of this phenomenon further in detail.

### Neuron type differences in neural decoding accuracy

Neurons in NCM separated nicely into narrow and wide spike neurons based on their spike waveforms. In both Experiments 1 and 2, single-unit populations consisted of comparable numbers of the two neuron types, whereas previous studies reported narrow to wide spike neuron ratios ranging from ~1:4 to ~1:2 in NCM [41–44]. These discrepant findings might be due to the differences in the recording techniques or the spike-sorting algorithms used in this and other studies. In the mammalian cortex, it is shown that narrow spike waveforms indicate inhibitory neurons and wide spike waveforms indicate excitatory neurons [45]. If this formulation is correct in the songbird forebrain, then the equal proportions for the two neuron types found in this study would be a more accurate depiction of the underlying circuitry since both histological [46] and functional [47] analyses showed that roughly half of the neurons in NCM are GABAergic inhibitory neurons.

Narrow spike neurons in NCM had higher baseline firing rates and stronger stimulus-driven responses compared to wide spike neurons, as shown in previous studies [42–44]. In parallel, intracellular recordings in brain slices from NCM indicated that half of the neurons fire phasically, whereas the other half fires tonically or transiently in response to electrical stimulation [48]. Importantly, the spike waveforms of the phasic neurons are wider than those of the tonic and transient neuron types. Unfortunately, those authors did not examine whether these differences in firing properties and spike waveforms reflected an excitatory/inhibitory neuron type differentiation. The results of the present study also revealed a marked difference between the adaptation profiles of the two neuron types. Wide spike neurons underwent strong adaptation with repeated stimulus presentation, whereas there was little or no adaptation for narrow spike neuron responses. A similar effect was shown by comparing the magnitude of adapted responses between the two neuron types in anesthetized Bengalese finches [43]. In the mammalian primary auditory cortex, two types of inhibitory interneurons are shown to contribute differently to the stimulus-specific adaptation (SSA) of excitatory neuron responses in the oddball paradigm [49]. Parvalbumin-positive neurons provide a global inhibition, whereas somatostatin-positive neurons inhibit excitatory responses to only the frequently repeated stimuli. The net effect of these two inhibitory processes is to adapt the excitatory neuron activity to repeatedly presented stimuli. If the narrow and wide spike neurons in NCM reflect inhibitory and excitatory neurons, respectively, then the strong adaptation observed for wide spike neurons might potentially be explained by similar circuit interactions. However, to date, the representation of parvalbumin- and somatostatin-positive interneurons has not been well-characterized in songbird NCM. Future studies utilizing *in vivo* imaging, together with cell type-specific optogenetic manipulations, are needed to address whether the long-term form of adaptation in the songbird NCM is an emergent property of such complex interactions between excitatory and different types of inhibitory neurons.

In both Experiments 1 and 2, narrow spike neurons yielded higher neural decoding accuracy and mutual information estimations than did wide spike neurons. Previous studies reported that wide spike neurons display higher levels of stimulus selectivity compared to narrow spike neurons in the starling general auditory lobule [41] and, more specifically, in the zebra finch NCM [42,44]. This might seem in conflict with the present findings, however the selectivity measures used in the mentioned studies were based on total firing rates as opposed to the neural decoding method based exclusively on the temporal profiles of responses used throughout this study. Indeed, when temporal neural codes were used, higher levels of stimulus discrimination and decoding accuracy was observed for narrow compared to wide spike neurons in the zebra finch auditory lobule [50] and, more specifically, NCM [51]. Despite the differences between narrow and wide spike neurons in overall mutual information, the two neuron types showed similar improvements in neural discrimination with repeated stimulus presentation. This suggests that the improvement in neural decoding performance observed in multi-unit responses with stimulus familiarization does not stem from differential contributions of the two neuron types, but rather occurs similarly at the single neuron level for both kinds of cells.

### Conclusion

This study provides valuable insights into the mechanisms by which the nervous system dynamically modulates sensory representations to improve discrimination of external signals at short and long timescales. When stimulated with auditory signals that have never been heard before, neural representations are rapidly modulated with just a few exposures to dramatically improve recognition and discrimination between complex sounds. During subsequent exposures, these signals are then successfully recognized after hearing fewer initial acoustic features. In addition, the nervous system can better recognize and discriminate these sounds when they are encountered one-by-one in a blocked order as compared to in an unpredictable sequence. The rapid plasticity in neural representations of novel auditory signals not only affects immediate processing, but is also long-lasting. That is, the discrimination of previously familiarized sounds is improved and occurs faster as compared to discrimination of completely novel signals. Taken together, these findings shed light on how the adult sensory system retains neuroplasticity that enables the organism to rapidly encode and classify sensory signals in an ever-changing world. Similar mechanisms may also be engaged during processing of human speech signals, and thus have a significant potential translational relevance to understanding the neural underpinnings of speech perception and comprehension difficulties.

## Materials and methods

### Subjects

A total of 32 naïve adult (>120 days) male zebra finches (*T*. *guttata*) purchased from a commercial supplier (Magnolia Bird Farm, Anaheim, CA) were used in this study. All birds lived in same-sex cages in a general aviary (LD 12:12, 21–25 Cº) with *ad libitum* food and water until the beginning of the experiments. All procedures were approved by Rutgers University Institutional Animal Care and Use Committee (Protocol Number 02–217).

### Surgery

Prior to the beginning of the experiments, birds underwent a surgery under isoflurane anesthesia (2–3% in oxygen; Henry Schein Animal Health, Dublin, OH) and placed in a stereotaxic apparatus. A craniotomy was performed over the region of interest and a metal pin was attached anterior to this opening with dental cement (Dentsply Caulk, Milford, DE) to be used to fix the bird’s head during subsequent electrophysiological recordings. All birds were injected with meloxicam (0.01 ml of 5 mg/ml; Boehringer Ingelheim, Ingelheim am Rhein, Germany) at the end of the surgery and recovered within an hour.

### Electrophysiology

Awake, restrained electrophysiological recordings were conducted in a walk-in sound attenuation chamber (Industrial Acoustics Company, Bronx, NY). Two silicon probes (NeuroNexus, Ann Arbor, MI), one for each hemisphere, were used for recordings. Each probe included 16 recording sites (0.4–1 MΩ impedance at 1 kHz) in a 4-by-4 grid layout. The probes were implanted in a para-sagittal plane such that the 4-by-4 grid layout extended in anterior-posterior and dorsal-ventral axes. Each probe was used for the right hemisphere in half of the birds and for the left hemisphere in the other half for each experimental condition. Prior to insertion, the probes were dipped into a DiI solution (10% in ethanol; Sigma Aldrich, St. Louis, MO) and allowed to dry to label probe insertion tracks for later histological analyses. The dura mater was opened and the probes were placed on the surface of the brain, above NCM in each hemisphere according to stereotaxic coordinates. Then, the probes were lowered by means of hydraulic microdrives, while playing zebra finch songs that were different from those to be used as experimental stimuli. When firing patterns characteristic of NCM neurons were observed at the majority of the recording sites, the experiment started. Multi-unit neural recordings were high- and low-pass filtered (0.3 and 5 kHz), amplified (10,000x), digitized (25 kHz), and saved to disk using Spike2 software (Cambridge Electronic Design, Cambridge, UK).

### Stimulus Presentation

#### Experiment 1

The experimental stimulus set consisted of 8 male zebra finch songs (1 motif each) selected from a corpus of recorded vocalizations that the experimental birds had never heard before. The acoustic similarities between all pairs of stimuli were calculated using Sound Analysis Pro software [52]. Briefly, four different acoustic features—pitch, frequency modulation, spectral continuity, and Wiener entropy—were calculated for each stimulus pair, a probability-based goodness of the match measure was estimated for each of these features between the two sounds, and these estimations were finally integrated into a global percent similarity score. The experimental stimuli were selected from the corpus such that all pairwise acoustic similarity scores were within the typical range with no outliers. This resulted in pairwise percent similarity scores between 39% and 63% (Mean ± SD = 51 ± 6%). The durations of the experimental stimuli ranged from 658 to 825 ms (Mean ± SD = 745 ± 49 ms).

Birds were randomly divided into two groups based on the type of stimulus presentation sequence with which they would be presented during electrophysiological recordings. For 8 birds, the experimental stimuli were presented in a blocked sequence, whereas for the other 8 birds, a shuffled sequence was used. During electrophysiological recordings, each stimulus was played 25 times at an onset-onset ISI of 6 s from a speaker located 30 cm in front of the bird at an amplitude of 55 dB SPL (A scale) and a sampling frequency of 44.444 kHz. In the blocked sequence, the order of stimulus blocks was pseudorandomly counterbalanced across birds such that each stimulus occurred in each position once.

#### Experiment 2

Two different stimulus sets were used in Experiment 2. The experimental stimulus set consisted of the 8 zebra finch songs described for Experiment 1. The control stimulus set included 8 other zebra finch songs, also selected from the same corpus of unfamiliar vocalizations, such that the percent acoustic similarity scores between the control and experimental stimuli ranged from 36% to 65% (Mean ± SD = 52 ± 6%). The durations of the songs in the control set were between 652 and 840 ms (Mean ± SD = 781 ± 54 ms).

Birds were randomly divided into two groups based on the stimulus set to which they would be passively exposed prior to electrophysiological recordings. The 8 birds in the pre-exposed group were presented with the experimental stimulus set that would also be used during subsequent electrophysiological recordings, whereas the 8 birds in the control group were presented with the control stimulus set. Note that the control group was also tested with the experimental stimulus set during electrophysiological recordings. Passive auditory exposure was conducted individually in the walk-in sound attenuation chamber also used for electrophysiological recordings. During auditory exposure, the birds were housed in a cage, but were not head-fixed. Each stimulus was played 200 times in a shuffled sequence at an onset-onset ISI of 6 s from a speaker located 70 cm in front of the cage at an amplitude of 60 dB SPL (A scale) and a sampling frequency of 44.444 kHz. At the end of the auditory exposure, birds were left isolated in the sound attenuation chamber until the beginning of electrophysiological recordings.

Electrophysiological recordings of neural responses to experimental stimuli started 20 h ± 15 min from the beginning of the passive auditory exposure. Since the passive auditory exposure lasted for 2 h 40 min, there was ~17 h 20 min from the last stimulus pre-exposure to the beginning of electrophysiological recordings. All birds were tested with the experimental stimulus set to which the pre-exposed group, but not the control group, had been exposed. All other stimulus presentation parameters were as described for Experiment 1, except that only the shuffled stimulus presentation sequence was used.

### Histology

At the end of the electrophysiological recordings, birds were deeply anesthetized with an overdose of pentobarbital (0.15 ml of 39 mg/ml; Vortech Pharmaceutical, Dearborn, MI), transcardially perfused with saline (0.9%, 40 ml) and paraformaldehyde (4%, 40 ml), and decapitated. The brains were extracted and post-fixed with paraformaldehyde for at least 4 days, after which 50-μm sagittal sections were cut on a vibratome. Unstained sections were visualized under a fluorescence microscope and grayscale digital images of the same sections were collected under 450-490/515-cut-on and 510-560/590-cut-on nm excitation/emission filters for anatomical markers and DiI, respectively. Two images from the same sections were superimposed to create composite images, and scaled drawings of the silicon probes were used to validate the recording sites that fell within the boundaries of NCM. Only recording sites that were at least 200 μm posterior of field L, which can be clearly identified by its cytoarchitecture, were included in data analyses (**Fig 1A**).

### Data analysis

Raw neural recordings were visually assessed by a human operator and trials with movement artifacts were excluded. Then, multi-unit spiking activity at each recording site was thresholded at 2 standard deviations from the mean amplitude (calculated from the whole recording) and the peaks of positive threshold-crossings were marked with timestamps, each representing a spike, with a time window of 1.24 ms (**Fig 1B**). In addition to these multi-unit spike trains, single-unit spike trains were also extracted by spike-sorting the raw neural recordings via an unsupervised technique as described in [27]. From the resulting single-unit clusters, only units with < 2% of their spikes within a 2 ms refractory period and with more than 2000 spikes throughout the entire recording were included in the final data set. All further analyses were carried out using custom scripts in MATLAB (The Mathworks, Natick, MA) and SPSS (IBM, Armonk NY) software.

#### Response magnitude

To quantify the magnitude of stimulus-driven neural responses, the firing rate during each baseline period (500 ms window preceding each stimulus presentation, *FR_base_*) and each stimulus period (stimulus duration period plus 100 ms, *FR_stim_*) was calculated as spikes/second. The response magnitude for each presentation of each stimulus was calculated as

$$Response\;Magnitude={FR}_{stim}-\bar{{FR}_{base}}$$

where $\bar{{FR}_{base}}$ is the average of the baseline firing rates across all repetitions of that particular stimulus. To control for between-stimulus and between-unit variability, the response magnitudes to all presentations of a given stimulus were calculated as a percent of the response magnitude to the first presentation of that particular stimulus.

#### Adaptation rate

To quantify the rate of adaptation of neural responses, stimulus presentations 1 to 6 and 6 to 25 were analyzed separately as in previous studies [17,26]. For each of these two sets of presentations, a linear regression analysis between stimulus presentation numbers and raw response magnitudes was conducted for each stimulus separately. The adaptation rate for each stimulus was calculated as

$$Adaptation\;Rate=100\;(b/\bar{RM})$$

where *b* is the slope of the linear regression and $\bar{RM}$ is the average of the raw response magnitudes on the set of stimulus presentations that was used for that analysis. This adaptation rate metric provides a normalized measure of adaptation, enabling comparisons across stimuli and units with varying average response magnitudes.

#### Neural dissimilarity

To calculate the dissimilarities between the temporal profiles of different neural responses, the spike counts during the stimulus-evoked response period were first grouped into 10-ms bins since peak mutual information estimations in NCM are seen at 5 to 10-ms temporal resolutions [15]. The duration of the response period for all stimuli was taken as 750 ms, which was equal to the minimum stimulus duration plus 100 ms. To develop a dissimilarity metric that is only sensitive to the temporal profiles, but not to the total firing rates, neural responses were standardized via taking the z-score of each bin by normalizing it with the average and the standard deviation across all bins within the same trial. Then, neural dissimilarity was quantified by calculating the Euclidean distance between these z-scored response profiles of the same unit to different pairs of stimulus presentations as

$$Neural\;Dissimilarity=\sqrt{\sum_{i=1}^{n}{\left(A_{i}-B_{i}\right)}^{2}}$$

where *A* and *B* are the binned response profiles in the two stimulus presentations and *n* is the number of bins. Similar Euclidean distance-based metrics have been widely used as measures of spike train dissimilarity [53]. The pairwise dissimilarities between each presentation of a particular stimulus and all presentations of all of the other stimuli were averaged to calculate between-stimulus neural dissimilarities. Similarly, the pairwise dissimilarities between each presentation of a particular stimulus and all of the other presentations of the same stimulus were averaged to calculate within-stimulus neural dissimilarities.

#### Neural decoding

Neural dissimilarity calculations described above were used to decode stimulus identities from the temporal profiles of neural responses. The dissimilarities of a particular focal response to the responses on all presentations of each stimulus were averaged, which produced 8 average neural dissimilarities, one for each of the 8 stimuli. The focal response was assigned to the stimulus with the minimal average neural dissimilarity. To assess the decoding performance from stimulus onset to any given point along the stimulus duration, this decoding procedure was conducted by progressively increasing the number of bins that went into the calculation starting from the stimulus onset. That is, for the first bin, the decoding was based solely on the neural responses in the first bin; for the second bin, the decoding was based on the neural responses in the first and second bins; for the third bin, the decoding was based on the neural responses from the first to the third bin, and so on. For decoding at each bin, the probability of correct decoding was calculated by counting how many of the 8 stimuli were correctly classified for a given stimulus presentation. The chance level for correct decoding probability was 1/8 = 0.125. In addition to the correct decoding probabilities at selected time points, the latencies along the stimulus duration to reach specific probability levels were also analyzed. To do this, correct decoding probabilities within each trial were linearly interpolated from the 10-ms to the 1-ms temporal resolution and the time point at which the specified probability level was first reached from the beginning of the stimulus presentation was taken as the latency. If the correct decoding probabilities never reached to the specified level, then the latency was taken as 750 ms, which represents the end point of the stimulus duration.

#### Mutual information

The decoding procedure described above was further used to calculate the mutual information between stimulus identities and temporal profiles of neural responses. For decoding at each bin, the true and the neurally decoded stimulus identities were used to construct a confusion matrix, from which mutual information was calculated using Shannon’s formula as

$$Mutual\;Information=\sum_{s,r}p(s,r){log}_{2}(\frac{p(s,r)}{p(s)p(r)})$$

where *s* is the true and *r* is the neurally decoded stimulus identity. The multiplication 0*log2(0) was equated to 0 and the prior probability *p*(*r*) for each stimulus was taken as 1/8 = 0.125, since all 8 stimuli were presented an equal number of times. The maximum possible mutual information was log_2_8 = 3 bits. To correct for the bias in mutual information estimations, for every unit, the calculation above was repeated 5 times while randomly shuffling the stimulus-response relationships across trials for each iteration. The mean across these 5 mutual information calculations was taken as the bias and subtracted from the real estimates so that bias-corrected mutual information estimates were used for all statistical analyses.

#### Slopes of the neural discrimination metrics

The directions and rates of changes in neural discrimination metrics with repeated stimulus presentation were quantified using linear regression and normalization methods as described for adaptation rates. These calculations were conducted for between-stimulus and within-stimulus neural dissimilarities, as well as correct decoding probabilities and latencies, separately. Trends for presentations 1 to 6 and 6 to 25 were analyzed separately in conjunction with the analysis of adaptation rates.

#### Spike waveform clustering

Following previous studies, single-units were divided into two clusters based on their spike waveforms. Single-units from the two experiments were pooled together with single-units recorded using the same recording system and stimulus presentation paradigm in other experiments to increase the sample size for more accurate clustering results. The average waveform of each single-unit was first normalized by its peak amplitude. Then, all waveforms were time-aligned by their positive peaks and processed via a principal components analysis. The first two components were used in an affinity propagation clustering algorithm [28] to classify waveforms into two clusters (**Fig 8**). Similar to previous reports [41], this method nicely separated single-units into narrow and wide spike neurons.

#### Statistical analyses

Parametric statistical tests were used for the analysis of multi-unit response measures, because they did not deviate significantly from normality. Factorial models with interactions were assessed via ANOVAs, and post-hoc comparisons were conducted via independent- and paired-samples t-tests with Bonferroni-corrected alpha values. For testing the difference of samples from single values, one-sample t-tests were used and, for testing the strength of pairwise relationships, Pearson’s correlations were calculated. Single-unit response measures, on the other hand, deviated severely from normality, thus nonparametric statistical tests were used for single-unit analysis. Due to a lack of nonparametric ANOVA for assessing factorial designs, Mann-Whitney U and Wilcoxon signed-rank tests were used for between-subjects and within-subjects comparisons, respectively. In addition, a one-sample Wilcoxon signed-rank test was used for testing the difference of a sample from a single value.
